# The Diagnostic Elimination Diet and Challenge Protocol for Management of Patients with Food Intolerance—Purpose and Development

**DOI:** 10.3390/nu18193220

**Published:** 2026-09-29

**Authors:** Anne Swain, Jo Ann Malinao, Velencia Soutter, Robert Loblay

**Affiliations:** 1 Department of Clinical Immunology & Allergy, Royal Prince Alfred Hospital, Camperdown, NSW 2050, Australia; joann.malinao@health.nsw.gov.au; 2 Department of Nutrition & Dietetics, Royal Prince Alfred Hospital, Camperdown, NSW 2050, Australia

**Keywords:** elimination diet, challenge, food intolerance, salicylate, biogenic amine, glutamate, preservative, color, wheat, gluten, milk

## Abstract

**Background**: In the 1960s and 1970s, several authors reported that manipulation of diet by excluding certain additives and natural chemical substances led to apparent clinical benefit for patients with adverse food reactions involving the skin, gastrointestinal tract, respiratory tract, and central nervous system. **Methods**: Over the past 48 years, patients of all ages presenting to the Royal Prince Alfred Hospital Allergy Unit with adverse food reactions were offered an elimination diet low in natural salicylates, biogenic amines, and glutamate, and free of additives such as preservatives and colors, for two to six weeks. Those who became asymptomatic were offered testing with a “N-of-1” double-blind, placebo-controlled (DBPC) challenge protocol. **Results**: There were 14,119 patients who elected to undertake either the strict elimination diet (10,074) (71.4%), the moderate approach (2760) (19.5%), or the simple approach (1285) (9.1%). Of these, 6645 (47.1%) became asymptomatic within two to six weeks; 628 (4.4%) reported no improvement; and 6846 (48.5%) were lost to follow-up. Of those who improved, 4963 (35.2%) returned for interpretation of their challenges, and 2429 had undertaken open food challenges; 3326 had completed the DBPC capsule challenge protocol, and 1830 completed both DBPC capsule and food challenges. Although individual reactivity was idiosyncratic, a highly reproducible pattern was evident across the entire group. The proportion reacting to each of the active chemical challenges versus placebo was highly significant (*p* < 0.001). **Conclusions**: In many patients with food intolerance, idiosyncratic dietary triggers can be reliably identified with a standardized elimination diet and DBPC challenge protocol. Long-term symptom control can be achieved by appropriate dietary modification.

## 1. Introduction

### 1.1. Food Intolerance Is Challenging to Manage

Food intolerance is one of medicine’s modern enigmas [1]. Its etiology and mechanism are unclear and the subject of constant debate [2], while estimates of its prevalence vary widely from 2% to over 20% of the population [1,3]. Intolerance symptoms also vary from person to person (idiosyncratic) and can change or fluctuate throughout their lifespan. Further, symptoms can have debilitating effects on the quality of life for individuals—personally, socially, and financially [4,5]—leading to significant societal costs in terms of loss of work/school days and increased pressure on an already over-burdened healthcare system [6].

For clinicians managing patients who present with adverse reactions to food, food intolerance continues to be a challenging area of professional practice [3,7,8,9,10,11,12,13]. Lifestyle, work, family, cultural and personal preferences, socioeconomic factors, and social networks are all relevant matters that make diagnosis challenging and need to be considered for successful management of patients [5].

Individuals with conditions requiring special diets may experience several psychosocial challenges due to the nature of their symptoms and the burden of treatment [14,15,16,17]. When prescribing dietary therapies, it is important to consider any history of disordered eating, orthorexia symptoms, avoidant/restrictive food intake (ARFID), food anxiety, and conditioned food aversions (fear and/or avoidance of specific foods that a person associates with a negative outcome) [18,19]. Food-intolerance symptoms can contribute to food anxiety, causing over-elimination of too many foods and/or entire food groups, particularly when patients suspect issues with multiple classes of foods (e.g., Fermentable Oligosaccharides, Disaccharides, Monosaccharides, and Polyols (FODMAPs); high histamine; and salicylates) [20]. Accordingly, psychosocial support can be important in managing the patient [21,22].

Providing adequate nutrition to patients when testing for food intolerance and prescribing long-term management to alleviate symptoms may also be challenging. The nutritional goal is always to provide the least restrictive and most varied diet modification which minimizes symptoms and optimizes diet quality [16,23]. It is recognized that a correct diagnostic approach and dietary control of food intolerance can minimize the use of inappropriate diets with severe nutritional deficiencies, thus helping to improve patients’ quality of life and reduce the economic costs of their management [24].

Identifying offending chemicals is crucial for managing a patient’s symptoms [25,26]. However, foods vary tremendously in chemical composition. The tastier a food is, the richer it is likely to be in a number of natural chemicals [8]. Although people are usually correct if they think their symptoms are diet-related, they often incriminate the wrong components based on what they mistakenly believe to be the common denominators. A variety of unvalidated tests have been widely promoted but are not reliable for diagnosis of food intolerances. Numerous costly alternative diagnostic methods for suspected food intolerances can be found online and are sometimes promoted to physicians or used by practitioners of complementary and alternative medicine [27,28,29]. These include breath tests, blood tests for serum IgG or IgG4 food antibodies, finger-prick tests (cytotoxic tests and “live blood” assay), laser probe fingertip testing, hair testing, and applied kinesiology testing [27,28,29]. These tests often lack well-designed controlled trials confirming their efficacy and have the possibility of leading to incorrect diagnoses, unnecessary elimination diets, and adversely altering the quality of life for patients and families [28]. Competing and contradictory understanding of food chemicals and food intolerance, as well as widespread use of unproven diagnostic tests, add to the complexity of clinical management of food intolerance.

### 1.2. Differentiating Between Food Allergies and Food Intolerances

Understanding the difference between intolerances and other types of food reactions, such as food allergies, is an important distinction as the clinical approach to dealing with each of them is quite different [30,31,32,33].

**“Food allergies”** occur in “atopic” people—those born with an overactive immune system, which, when stimulated by exposure, is genetically predisposed to produce high levels of “IgE” antibodies against otherwise harmless protein substances (“allergens”) in the diet and/or environment. In general, the proteins responsible for particular food allergies are unique to that specific food (for example egg, milk, peanut, tree nuts, fish, seafood, wheat, and soy), and dealing with them involves identifying and avoiding all traces of that food [34,35]. Typically, “allergic sensitization” to food allergens occurs during infancy and early childhood, and there is a tendency for many of them to resolve naturally as children grow older. However, failure to resolve by the late teens (especially nut or seafood allergy) is usually associated with life-long persistence [10,36,37,38].

**“Food intolerances”** are adverse food reactions that, unlike food allergies, do not involve the immune system [31,32]. In most cases, food intolerance reactions are triggered by food chemicals that cause symptoms by irritating nerve endings in different parts of the body, similar to the way that certain drugs can cause side-effects in sensitive people [2,39]. Some people are born with a sensitive constitution which reacts more readily to food chemicals. This tendency is probably inherited, but environmental triggers—a sudden change of diet, a bad food or drug reaction, or a nasty viral infection (for example, gastroenteritis and glandular fever)—can bring on symptoms at any age by altering the way the body reacts to food chemicals. Women often become more sensitive in their child-bearing years, perhaps due to hormonal changes, which might be nature’s way of preventing pregnant and breast-feeding women from eating foods that could harm a developing baby [39,40]. Babies are more vulnerable to food chemicals because their metabolism, and gastrointestinal and nervous systems are immature, leading them to prefer bland foods. As children mature, their bodies become accustomed to handling more of the rich, spicy, and highly flavored foods enjoyed by the rest of the family [41,42].

Sensory nerves can be stimulated by a range of external chemicals that we are exposed to in the diet and environment. These may be pleasurable sensations or unpleasant symptoms, depending on the nature of the stimulus, our genetic background, past experiences, and the current sensitivity settings of our nervous system [2,39].

### 1.3. Substances Most Likely to Provoke Intolerance Reactions

Chemicals are found everywhere in nature, including in foods. Some can be beneficial in small amounts—for example, the vitamins we need for good health—and the flavor and aroma substances that make food so enjoyable [43]. On the other hand, many plants contain substances that are poisonous to humans, and, of course, we avoid cultivating these as foods [44,45]. The staple foods we eat today have been selected by trial and error over thousands of years, both for their nutritional value and because most people can tolerate the low levels of natural chemicals present without getting sick. However, even these can cause illness if excessive amounts are consumed. This is the main reason for recommending that people eat a varied diet and have all foods in moderation [46,47].

The natural substances most likely to upset sensitive individuals—salicylates, biogenic amines, glutamate, wheat, and dairy—are the ones common to many different foods and are therefore consumed in greatest quantity in our daily diet [3,7,8,9,10,11,12].

***Salicylates*** are a family of natural chemicals produced by all plants for defense against insects, pests, and microbial attacks. High levels are found in many fruits, vegetables, nuts, herbs and spices, jams, honey, yeast extracts, tea, coffee, juices, beer, and wine [48,49,50,51,52,53,54,55]. They are also present in natural flavorings (e.g., mint and fruit flavors) used in foods, drinks, and liquid medications, and as volatile substances in perfumes, toiletries, cleaning products, washing powders, and botanical oils (e.g., lavender, eucalyptus, tea-tree, and wintergreen) [56,57,58].

***“Biogenic” amines*** are naturally present in animals and plants, where they act as neurotransmitters, hormones, and inflammatory mediators. Increased levels can develop in foods as a result of protein breakdown due to bacterial action as the foods age or “mature” (e.g., meats, fish, and cheese), from high-temperature cooking (e.g., grilling and frying), and with the ripening of fruits (e.g., bananas, tomato, avocado, pawpaw, and olives). High levels are also present in sauces, fruit juices, chocolate, jams, flavored spreads, and nut seed pastes, and in fermented products (e.g., beer, wines, and yeast extracts) [59,60,61,62,63,64,65,66].

***Glutamate*** is an amino acid building block present in all proteins. Anytime food is aged, fermented, or cooked for many hours, there will be increased levels of free glutamate—not linked to proteins—enhancing the flavor of the food. This is why foods rich in free glutamate (such as tomato, cheese, and stock cubes) are used in many meals, and why pure monosodium glutamate (MSG) is added to noodles, soups, sauces, and many dishes in Asian cooking, creating the unique taste of umami [67,68,69,70,71,72,73,74,75,76].

***Food additives***: People who are sensitive to natural food chemicals are often also sensitive to one or more of the common food additives, such as preservatives, artificial colors, and/or flavorings. Reactions to these can be easier to recognize than reactions to natural chemicals because the higher doses present in processed foods trigger more immediate symptoms, making the cause-and-effect relationship more obvious [77,78,79,80,81,82,83,84].

***Gluten/wheat***: Many people with an irritable bowel experience improvement with gluten avoidance and find that eating wheat causes a relapse of symptoms, even though they do not have celiac disease. This has come to be known as “non-celiac gluten intolerance”, but the mechanism is not known [85,86,87,88,89,90,91,92]. However, experience shows that non-celiac gluten intolerance is rarely an isolated problem [93,94,95]. If gluten is identified as a problem, it is very likely that they also have unrecognized intolerances to one or more of the natural food chemicals, milk and/or soy [27,32].

***Milk/dairy***: Many people with an irritable bowel improve when avoiding dairy or dairy products without having a lactose intolerance. This usually is not an isolated problem [96,97,98,99,100]. If milk triggers symptoms, other unrecognized intolerances are likely to be present, including wheat/gluten and natural food chemicals [27,32].

### 1.4. Common Clinical Manifestations of Food Chemical Intolerances

The most common symptoms are disorders of gut–brain interaction (DGBI), nausea, headaches, chronic spontaneous urticaria and angioedema (CSU/AO), and reflux (with heartburn, throat irritation, and/or sinus trouble) [3,10,12,27,32,39,101].

Children can become irritable and restless, and behavioral problems can be aggravated in those with nervous system disorders such as ADHD (attention-deficit/hyperactivity disorder) [10,12,84,102,103,104,105,106,107]. Even breast-fed babies can have reactions because of chemicals from the mother’s diet being in the breast milk, causing reflux, colicky irritable behavior, loose stools, eczema, nappy rash, and spotty rashes around the mouth [108,109].

The nature, onset, duration, and severity of reactions will depend on several factors:

- The symptoms a person is prone to;
- The chemicals a person is sensitive to;
- The degree of sensitivity to each chemical;
- The daily dietary intake of each chemical consumed from all food sources.

The timing and severity of reactions can vary too. Symptoms can begin within an hour or two or may be delayed by several hours or days. Mild reactions can be brief—a few hours—but more severe ones can sometimes continue for several days [3,24,32,39,110,111].

### 1.5. Factors That Determine the Nature of Food-Intolerance Reactions

#### 1.5.1. Dose Dependence

Food-intolerance reactions are dose dependent [112,113,114]. In other words, they depend on the amount and frequency of the particular chemicals an individual is sensitive to ingested daily from all food sources [106,115,116,117].

In affected individuals, intolerance reactions exhibit a dose threshold [118,119,120,121]. In people who are less sensitive (i.e., those with a high dose threshold), symptoms might only develop after a particularly rich meal or after bingeing on fruits, juices, and/or processed foods. Having a small amount of a chemical-rich food—for example, one or two strawberries or a slice of fresh tomato—may cause no noticeable symptoms. However, a larger amount—for example, eating a whole punnet of strawberries or having concentrated tomato as a sauce or paste—can provoke a reaction. In such cases, formal dietary testing for food-chemical intolerances may be unnecessary, since a simple reduction in intake of these foods may be all that is needed for prevention of recurrent symptoms [39].

#### 1.5.2. Cumulative Effects

In some people, eating small amounts (“sub-threshold” doses) of certain foods on a regular basis can have a cumulative effect—with a delayed onset of symptoms which can build up in frequency and intensity over several days. In these circumstances, the cause–effect relationship between diet and symptoms may not be obvious to the person concerned [39,116,122].

### 1.6. Different Approaches to Managing Food Intolerance

It has been known since the turn of the 20th century that aspirin ingestion could precipitate acute urticaria [123]. Calnan [124,125] noted that aspirin could also trigger recurrences in many patients with CSU/AO. During the 1960s and 1970s, several authors reported that a number of chemicals present in food could precipitate acute exacerbations of CSU/AO and that diets designed to exclude these substances often induced prolonged remission [112,113,114,126,127,128,129,130,131,132,133,134,135,136,137,138].

The implicated chemicals included the naturally occurring salicylates in food, as well as artificially added benzoate preservatives and colorings. Several of the above authors reported that diets designed to exclude foods containing salicylates and/or additives induced prolonged remission of urticaria in those patients who had shown a positive response to oral challenge with these compounds [129,131,133,134,135,136,138]. However, available diets were largely empirical, based on observation and clinical experience.

More recently, dietary management approaches include elimination of various food groups (for example wheat/gluten, milk/lactose, yeast, sugar, nightshades, and “acid” foods) or particular dietary components (FODMAPs) [3,16,24,139,140,141,142,143,144].

Examples of some of these popular elimination diets are outlined below in Table 1.

### 1.7. The Diagnostic Elimination Diet and Challenge Protocol (DEDCP)

The DEDCP [108,109] was developed by the Royal Prince Alfred Hospital (RPAH) Allergy Clinic (Clinic) as a diagnostic and management tool to assist clinicians in managing patients with food intolerance, and to facilitate patient-centered care [195,196,197].

The DEDCP is only one of the useful clinical tools available to health practitioners to manage adverse food reactions in patients. The diagnostic strict elimination diet is not for all patients, as other treatment modalities, including less strict elimination diets (moderate and simple approach), are available that can successfully treat them depending upon their presentation and symptoms [108,109]. The strict elimination diet is particularly relevant for patients who present with complex symptoms and triggers.

Specific intolerances are idiosyncratic. The chemicals involved vary from person to person, and are found in many different foods, so the clinical management approach developed involves identifying the problem chemicals for each individual and reducing their daily intake of the groups of foods, all of which contain the same offending substance or substances.

An “N-of-1” randomized trial approach to dietary investigation [198,199,200,201,202,203,204,205,206], where each patient is their own control, proved to be the most appropriate diagnostic method especially for complex patients presenting with a variety of symptoms and triggers.

With this approach, the DEDCP has been developed and the capsule challenges refined over 48 years to be a thorough investigative method: (a) to identify offending foods and chemicals triggering reactions in an individual and (b) to develop a personalized diet and liberalization plan to minimize and control symptoms. This paper traces the development, methodology, and scientific basis of the DEDCP. It presents a 48-year retrospective observational cohort study which aims to demonstrate the clinical application of the DEDCP in patients who utilized the diet and challenge protocol for dietary management of their symptoms. It outlines the background, history, design, and changes to the challenge protocol over time.

## 2. Materials and Methods

### 2.1. Ethics

Ethical approval for this study was granted by the Sydney Local Health District Human Ethics Review Committee (Protocol Nos. X24-0430 and 2024/ETH02797).

### 2.2. Role of the Royal Prince Alfred Hospital Allergy Clinic

This study was undertaken by the RPAH Allergy Clinic [hereafter, the Clinic], which was established by Dr. R.L. Clancy in 1977 as a multidisciplinary Clinic. It included an immunologist, respiratory physician, dermatologist, and dietitian. This study commenced after Dr. Clancy became interested in the possible role of dietary substances in the pathogenesis of CSU/AO. As a result of the favorable findings reported by Warin and Smith [135], it was decided to investigate dietary management for treatment of CSU/AO. After reviewing the available literature on diet and CSU/AO [133,135,207,208,209,210,211,212], the elimination diet was formulated by the Clinic [213].

### 2.3. Development of the Diagnostic Elimination Diet and Challenge Protocol (DEDCP)

Building upon prior research, the Clinic commenced this study in April 1977 to develop a Diagnostic Elimination Diet and Challenge Protocol. This was designed to investigate the role of food in CSU/AO. The elimination diet was based mainly on the elimination diets of Rowe (1944, 1972) [208,210], Shelley (1964) [211], Feingold (1968) [212], and Warin (1976) [133,134,135] designed to exclude the common food “allergens”, as well as salicylates, preservatives, and azo dyes.

Stable asymptomatic remission was first established on this baseline diet, prior to challenge. Following completion of the challenge protocol, long-term dietary modification based on individual oral provocation results was provided to the patient.

The Clinic approach to the identification of dietary chemicals which contributed to the pathogenesis of CSU/AO was similar to that of Warin and Smith [135]. An important difference, however, was the attempt to establish a stable asymptomatic remission prior to challenge, using a baseline diet constructed to exclude all the test substances [213]. This approach had the dual advantage that patients would be able to discontinue medications (antihistamines), which might otherwise mask challenge reactions, as well as reducing background symptoms from substances included in a normal diet which might otherwise produce false-positive results. The low response rate with the placebo in this study emphasized the importance of removing this background “noise” and contrasted with the experience of other authors [129,138].

A detailed review of the literature revealed that, where available, analytical data on the content of salicylates and benzoates occurring naturally in foods was incomplete, scattered, and sometimes contradictory [110]. It was therefore decided to undertake a systematic thin-layer chromatography (TLC) and high-performance liquid chromatography (HPLC) analysis of the total salicylate content of commonly eaten foods [48]. With HPLC, a total of 330 foods were tested for salicylate content. This work led to a number of modifications of the original elimination diet (strict, moderate, and simple approach) [111,213].

In 1980, authors R.L. (Immunologist) and V.S. (Pediatrician) joined the Clinic. It became apparent that adverse reactions to food may cause not only symptoms involving the skin, but also of the gastrointestinal tract, respiratory tract, and/or central nervous system.

By 1982, the Clinic was well known for its work in food intolerance, and many patients were therefore referred with a variety of clinical problems, which they suspected might be attributable to dietary factors. These included headache/migraine [214,215,216,217,218,219,220,221,222,223,224]; disorders of gut–brain interaction (DGBI), i.e., irritable bowel syndrome (IBS) [225,226,227,228]; asthma and rhinitis [229,230,231,232,233,234,235]; eczema [236,237,238]; and symptoms referable to multiple organ systems, often together with vague constitutional symptoms suggestive of psychoneurosis, and a number of “hyperactive” children [239,240,241].

Clinical observations suggested that in patients with syndromes other than CSU/AO, milk [242,243,244,245], wheat [245,246,247], natural biogenic amines [214,216,217,248,249], MSG [250,251,252], and a variety of other food additives [128,253,254,255,256,257,258,259,260,261] might also be implicated in provoking some of these symptoms, and a stricter elimination diet was designed for the investigation of such patients. At the same time, the range of challenge substances was extended, and starch and sucrose were introduced as placebos in place of lactose, which was sometimes found to provoke abdominal symptoms.

Since many of these additional symptoms were of a subjective nature, the challenge protocol was made “double-blind”, with numbered challenge capsules administered in an arbitrary order [39,262]. In some patients, the double-blind, placebo-controlled (DBPC) capsule challenge was not practical or feasible, and, in these cases, open food challenges were carried out. This mainly occurred in (a) very young children who were unable to swallow the capsules or the powder, (b) patients who were terrified by the thought of taking capsules or their effect, or (c) patients who lived out of town without a close general practitioner who could supervise their challenges. Open food challenges consisted of specially selected foods according to chemical composition [110].

The DEDCP low in natural salicylates, biogenic amines, glutamates, additives, dairy and wheat/gluten, and DBPC capsule challenge and/or food challenge protocol has continued to be used for patients with adverse reactions to food. Responses to challenges are used to develop personalized diets for patients and a liberalizing plan to identify their dose-reaction thresholds to each compound [39].

A brief timeline outlining major milestones in developing the elimination diet and challenge protocol (strict, moderate, and simple) is shown in Appendix A. It also shows presenting symptoms that did not change over time and outlines publications and resources arising from the research.

### 2.4. Dietary Investigation and Management

Unlike food allergies, there are no skin tests or blood tests that can diagnose food intolerances, so a systematic trial-and-error method of dietary testing is necessary.

The “N-of-1” diagnosis of food chemical intolerances is comparable to the long-standing principle of elimination and challenge testing which has been the standard method for causality assessment in patients experiencing adverse drug reactions [198,199,200,201,202,203,204,205,206]. In using the “N-of-1” randomized trial method [198,199,200,201,202,203,204,205,206] of dietary testing, there are a number of points to keep in mind:

- It is not possible to predict from a person’s symptoms which particular food chemical(s) cause a reaction or sensitivity.
- The clinical history is often unreliable. Although people are usually correct if they think their symptoms are diet-related, they often identify or incriminate the wrong foods and/or food components.
- Most people with food chemical intolerances are sensitive to more than one substance.
- Attempting to eliminate foods or food chemicals one at a time often produces misleading results.

The only way of finding out which food chemicals may be contributing to symptoms is to eliminate all possible trigger substances at the same time, wait for symptoms to subside, and then reintroduce them one-by-one according to a systematic challenge method.

Appendix B diagrammatically illustrates application of the “N-of-1” trial principle in the diagnosis of food intolerances: (1) eliminate all potential (known) triggers from the diet; (2) challenge systematically (DBPC, or open food challenge); (3) reintroduce tolerated substances; and (4) liberalize diet to establish reaction threshold for each trigger substance.

We describe the methodology for the stepwise process of testing for intolerances below. This stepwise process was conducted as part of this study in which patients were invited to take part.

#### 2.4.1. Step One: Clinical and Dietary Evaluation

Each patient was assessed by a physician to exclude underlying systemic disease before undergoing dietary investigation [263]. All children were assessed by a pediatrician attending the clinic, and when psychosocial factors were felt to be the major problem, dietary investigation was not recommended. Patients expressing interest in diet were educated about food-intolerance principles and the DEDPC protocol.

Taking a detailed clinical history from each patient is very important. Patients completed a Diet Symptom Assessment Questionnaire (DSAQ) and Diet History Questionnaire (DHQ) (https://slhd.health.nsw.gov.au/rpa/services/clinical-immunology-and-allergy/supporting-our-patients) (accessed on 26 September 2026) designed to gather information about their symptoms, quality of life, known food triggers, past diets tried, and current dietary intake [7,9,10].

Individuals with a history of asthma underwent respiratory assessment before taking any challenges, which were administered under appropriate medical supervision (depending upon their degree of bronchial reactivity). Patients with a history of laryngeal edema and/or systemic anaphylaxis were routinely admitted to hospital for challenge.

#### 2.4.2. Step Two: Diagnostic Elimination Diet

Individuals with food intolerances vary in (a) their reaction to different foods and food chemicals, (b) their degree of sensitivity, (c) the frequency and severity of their symptoms, and (d) their personal dietary preferences and lifestyle. For these reasons, there is no one single diet that suits everybody.

Individual dietary recommendations were tailored to each person, considering their unique clinical symptoms, preferences, and lifestyle. Generally, the stricter the diet, the faster the symptoms settled and the quicker the offending foods and food chemicals were identified. In keeping with patient-centered care [195,196,197], individuals decided the level of diet they wanted to follow.

Appendix A and Appendix B outline three optional elimination diet approaches. We provide a summary of these approaches below.

***Strict elimination diet*** is for those with distressing symptoms that interfere with day-to-day life or work.

- Low in natural food chemicals—salicylate, biogenic amines, and glutamate [108,109,264];
- Avoids added preservatives, colors, and flavor enhancers;
- ±Wheat/gluten, milk/lactose, and soy milk;
- ±The “windy foods” (low-chemical, high-FODMAP foods) [265].

***Moderate approach*** is for those with less severe symptoms, and those who find the strict elimination diet too restrictive.

- Low and moderate in natural food chemicals—salicylate, biogenic amines, and glutamate [108,109,264].
- Avoids added preservatives, colors and flavor enhancers.
- ±Wheat/gluten, milk/lactose, and soy milk.
- ±The “windy foods” (low-chemical, high-FODMAP foods) [265].

***Simple approach*** is for those with infrequent or mild symptoms, where simple dietary changes are often sufficient to prevent symptom recurrences.

- Avoids very high chemical foods [108,109,264];
- Avoids added preservatives, colors, and flavor enhancers;
- ±Wheat/gluten, milk/lactose, and soy milk;
- ±The “windy foods” (low-chemical, high-FODMAP foods) [265].

The comprehensive food lists and charts are publicly available [108,109,264] and were considered too long to be included in the manuscript.

The elimination diet contained adequate protein, fat, and carbohydrates provided that the dietary recommendations were followed (https://www.eatforhealth.gov.au) [266,267]. However, the level of vitamin A was low due to restriction of a range of fruits and vegetables and milk/dairy foods, so the use of an uncolored, unflavored multivitamin was advised. All acetyl salicylic acid-based medications (Disprin, Alka-Seltzer, aspirin, and compound analgesics) were prohibited. Most flavorings used in toothpastes, cough lozenges, flavored medication, and syrups contain salicylate, and so alternatives were prescribed. Products containing wintergreen oil (Decorub and Deep Heat), a concentrated source of methyl salicylate, were forbidden as were some perfumes used to scent many toiletries because of amyl and benzyl salicylate [268,269,270].

Medications which were allowed included uncolored medications which did not contain acetylsalicylic acid, methyl salicylate, or their analogues. With other drugs, if suitable white medications were not available and therapy could not be interrupted, patients were instructed to wash the artificial coloring off the surface by rubbing gently under running water. Capsules could be opened if necessary, and the powdered contents (decanted into a clear capsule) taken, discarding the colored gelatine capsule.

Following confirmation of a diagnosis of CSU/AO, DGBI, IBS, aphthous ulcers, migraine, eczema, asthma, hyperactivity, or systemic symptoms by the physician, each patient was interviewed by the dietitian, and a detailed history taken. At this interview, the elimination-diet program was discussed with both the patient and the person responsible for preparing meals.

Patients were given information about food sources of natural salicylate [48], biogenic amines, glutamate, wheat/gluten, milk, and the artificial colors and preservatives. This gave each patient information as to the possible dietary precipitants of their symptoms. The importance of compliance was stressed, emphasizing that only those foods listed were to be eaten, as dietary infractions could lead to recurrence of symptoms and false positive or confusing challenge results. Detailed instructions and explanations were given about which foods to eat and which foods to avoid, along with the reasons for each inclusion and exclusion of food on the diet. Patients were warned that their attention to detail was critical to the success of the program.

Practical advice on shopping, food preparation, and how to vary the diet was given, as well as a list of recipes for preparation of palatable meals. The management of restrictions to lifestyle were also addressed. Detailed advice was given about lunches and snacks, takeaway food, eating out in restaurants, dinner parties, and other social occasions (weddings, parties, and sporting events), all of which provided practical information which was critical for patient compliance and confidence. At the same time, patients were advised about vitamin supplements, medications, cosmetics, and toiletries.

In this study, many patients followed a strict elimination diet for 2–4 weeks for symptoms to settle. The strict diet was to develop an asymptomatic baseline for challenge. It is a short-term diet, which excludes all foods containing chemicals that can trigger intolerance reactions.

Frequent contact with the dietitian over the telephone was necessary for encouragement and support. This included repeating dietary instructions and practical advice about day-to-day issues. It resulted in excellent compliance.

Patients kept a detailed food and symptom diary to keep a record of (a) all foods and beverages consumed, (b) the type and duration of all symptoms experienced, (c) details of all food and capsule challenges taken, and (d) the dose and type of medications taken when necessary.

Some experienced a brief flare-up of symptoms, or “withdrawal” effect. This can occur a week or so after commencing the strict elimination diet but usually subsides of its own accord after a few days [39].

Patients who experienced marked reduction (≥80% symptom improvement) or complete relief of symptoms for five consecutive days after a minimum of two weeks on the elimination diet were provided with challenge capsules which were sent by mail. Those with a history of laryngeal edema, anaphylaxis, or asthma were given their challenge in graded doses under appropriate medical supervision.

Based on clinical judgment, we selected ≥80% symptom improvement instead of a standard 30% to 50% reduction to ensure there was a high-level response or near-complete resolution of the symptoms and to demonstrate a successful clinical outcome, not just minor change. This was important as a clinical baseline for the challenges and to identify significant reaction response. This approach was a protocol-defined threshold based upon patient self-evaluation and physician/dietitian judgment.

If there was no significant improvement after two weeks, the diet was discussed with the patient to ensure that the instructions were understood and to check compliance. Patients who could not comply with the elimination diet for social, family, or other reasons did not undergo challenge.

For those patients who presented with CSU/AO, DGBI, IBS, migraine, eczema, asthma, hyperactivity, and systemic symptoms where there was no improvement after six weeks, continued restriction was not helpful. They were asked to return for review by the physician, and these patients were instructed by the dietitian as to how to gradually resume a normal diet. Foods were added back by chemical food groups every three days until a full diet was reached. A few patients experienced an exacerbation of their symptoms with these added foods groups. Patients with aphthous ulcers and eczema often took longer for symptoms to resolve, showing minimal improvement in the first six weeks. There was, however, continuing improvement over a further six weeks with marked improvement after 12 weeks. At that time, they were ready to be challenged.

#### 2.4.3. Step Three: Challenge Protocol and Testing

Once symptoms had settled for five consecutive days, a series of food challenges were conducted using foods specifically selected to contain a single food chemical and/or DBPC capsule challenges (Appendix C, Appendix D and Appendix E). This was conducted to identify substances triggering previously experienced symptoms.

The onset of challenge reactions can be delayed by several days. Salicylate and amine-containing food challenges should be taken for up to a week; glutamate and additive-containing food challenges for up to 3 days (Appendix F). A challenge should cease if symptoms develop.

Individual challenge reactions can last for several days before subsiding, following which, there can be a “refractory” period (lack of responsiveness to a subsequent challenge), so it is important to have at least 3 days free of symptoms before moving on to the next challenge. If challenges are rushed, patients are likely to get confused and may have to repeat the challenges, which will delay the diagnostic process.

- ***DBPC-challenge precautions*.** It is recommended that DBPC chemical-capsule challenges are used only in teaching hospitals. The chemicals and placebos can be encapsulated in opaque gelatine capsules and administered double-blind at 72 h intervals. (Appendix C, Appendix D and Appendix E).
- ***Individual patients with a history of asthma, laryngeal edema, and/or anaphylactoid reactions*** need to be in a hospital environment as inpatients or outpatients in a specialist clinic for challenge with salicylate, metabisulfite, and glutamate (Appendix D). The specialist clinic should have resuscitation facilities available.
- ***Individual patients with a history of asthma*** underwent respiratory assessment before taking challenges, which were administered under appropriate medical supervision (depending on their degree of bronchial hyperreactivity) (Appendix D) [39,111].
- ***Individual patients with a history of laryngeal edema and/or anaphylactoid reactions*** were routinely admitted to hospital for small-incremental-dose challenges with salicylate, metabisulfite, and glutamate (Appendix D).
- The elimination-diet and food-chemical challenges should be ***avoided in pregnant women*** because possible effects on the fetus are unknown.

##### Challenge Instructions

Patients were instructed to commence the challenge procedure of food and capsule challenges after at least two weeks on the elimination diet with five consecutive asymptomatic days. Open challenges with milk and wheat were taken first. These foods were added to the baseline elimination diet if there was no obvious reaction. Double-blind chemical challenges including placebos were then administered. Challenge capsules were taken in the morning half an hour before or two hours after breakfast (for children, the capsule contents were mixed with golden syrup or mashed potato if they were unable to swallow the capsules). The capsules were spaced by at least 72 h to allow for delayed reactions. A response to challenge was followed by a pause of a further three symptom free days before proceeding to the next challenge, since patients were often found to experience a temporary refractory period during which they are unresponsive to further challenge. Some challenges were divided into “A” and “B” doses, representing a small dose (“A”) and a larger dose (“B”) of the same or related compounds. These were taken on the same day, with the small dose (“A”) first, and if there was no reaction within two hours, the second dose (“B”) was taken. However, if a noticeable reaction occurred to dose “A”, the subsequent dose, “B”, was not taken.

##### Interpretation of Challenge Results

Challenges were initially given single-blind as a numbered sequence. However, by the end of 1981, both the physicians and the dietitian had become so familiar with the standard sequence that there was the danger of a biased interpretation with subtle reactions. Accordingly, in January 1982, the protocol was made double-blind, with the challenge substances being numbered in an arbitrary order for each patient.

After completion of challenges, patients were seen by the dietitian, who reviewed their diary and recorded the results. The dietitians were trained by the authors [R.L., V.S., and A.S.] in order to ensure patient uniformity of interpretation. A challenge was considered positive if there was a recurrence of symptoms within 48–72 h. Reactions were almost always clear-cut. Three reaction patterns were commonly observed: acute (onset half to two hours, duration one to two hours), intermediate (onset five to eight hours, duration up to 24 h), and delayed (onset 24–48 h, duration two to three days up to a week or more).

If the response to any challenge was uncertain, it was repeated as a capsule or by a three-to-seven-day open food challenge (Appendix F). This method provided a check when challenges were taken too quickly in succession due to the eagerness of the patient to complete the tests. Under these circumstances, overlap of challenges could lead to confusion about which one was responsible for a particular reaction. Conversely, a false negative could occur if the subsequent challenge was taken during the refractory period.

##### The Role of Dietitians

Systematic dietary testing is best undertaken with the assistance of a dietitian experienced in the diagnosis and management of food intolerances. They will be able to help interpret the patients’ challenge reactions, as recorded in their daily food and symptom diary. If there has been a lack of improvement after 4 weeks of dietary elimination, they will be able to help with “troubleshooting”, checking for inadvertent mistakes in adherence to the elimination diet, and checking that all potential non-dietary symptom triggers have been excluded. They can also perform a nutritional assessment if there are concerns about inadequate dietary intake during the testing period.

#### 2.4.4. Step Four: Personal Diet

A personal diet based on challenge results was developed, including foods containing chemicals that patients did not react to on challenge. Patients remained on their personal diet for a period of three to four weeks to double-check their challenge results.

#### 2.4.5. Step Five: Liberalizing Diet to Find Threshold(s)—Long-Term Dietary Management

Gradual liberalization of the diet by food chemical groups was encouraged to induce tolerance by raising the threshold for triggering symptoms. Patients were encouraged to liberalize the natural compounds to which they had shown sensitivity, i.e., natural salicylates, biogenic amines, and glutamate, by taking very small amounts on an intermittent basis and then increasing the frequency and amounts tolerated.

After 3–4 weeks on their personal diet, provided they remained well, foods containing chemicals that they did react to were systematically reintroduced, one group at a time, to find their dose-threshold for each food chemical. In patients with a mild degree of sensitivity, it was sometimes possible to return eventually to a near normal diet without relapse.

#### 2.4.6. Step Six: Living with Food Intolerances

Once patients had determined their dose threshold(s) for each problem chemical, they were able to adjust their diet and make a personal choice about how to balance the benefits of being free from distressing symptoms against the inconvenience of restricting their dietary choices. The aim was to develop a long-term diet that alleviated the patient’s adverse reactions and improved their quality of life.

## 3. Results

### 3.1. Study Population

Over the past 48 years, we have recorded symptoms and dietary information on 14,119 patients (2027 children (≤15 years old) and 12,092 adults (≥16 years old) who presented to the Clinic. Of the 14,119 patients, 3992 (28.3%) were male and 10,127 (71.7%) were female. Amongst the adults, there was a preponderance of females (3F:1M) compared with (1F:1M) in children. The presenting age of patients ranged from infancy to 97 years of age.

Not included are patients who presented to the Unit with conditions other than food intolerance. These patients were excluded to ensure that this study only involved patients with food intolerance, including patients presenting with food allergies, food protein-induced enterocolitis syndrome (FPIES), eosinophilic esophagitis (EoE), celiac disease (CD), and inflammatory bowel disease (IBD). Information for these patients in the database was incomplete in that it did not include all the information recorded for the food intolerance patients.

Patients were generally referred to the Clinic by their family doctor or a specialist; many came to see if changing their diet would eliminate or control their symptoms and reduce their need for medication. Patients presented with a range of symptoms (Appendix G) involving the skin (SKIN), gastrointestinal tract (GIT), respiratory tract (RESP), and central nervous system (CNS), as shown in Table 2.

Some patients presented with symptoms that were of one system (e.g., CSU/AO, DGBI, or headache), but a detailed clinical history and the Diet Symptom Assessment Questionnaire showed that they had symptoms involving more than one organ system, as shown in Table 2.

### 3.2. Food Triggers and Dietary Restrictions

Seven hundred and twenty-seven patients presented after an acute reaction to food. A significant number of patients N = 4372 (31%) had tried modifying their diet (e.g., Complementary, Alternative, Integrative Health Diets), as indicated in Table 3.

Patients presented (82%) with a list of foods, drinks, and other factors that they identified or suspected of causing their symptoms as shown in Table 4. 

**Figure 1:**
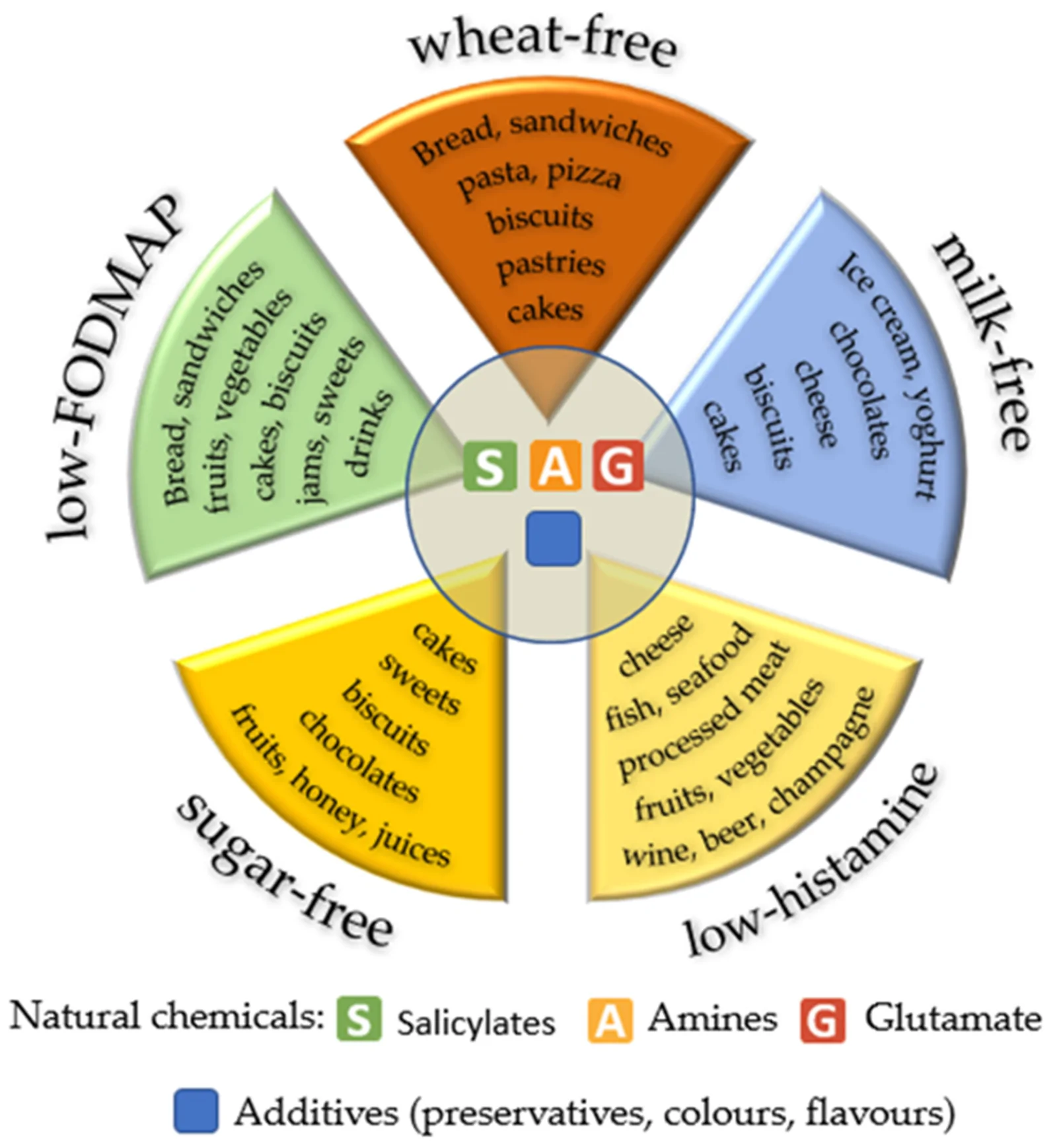

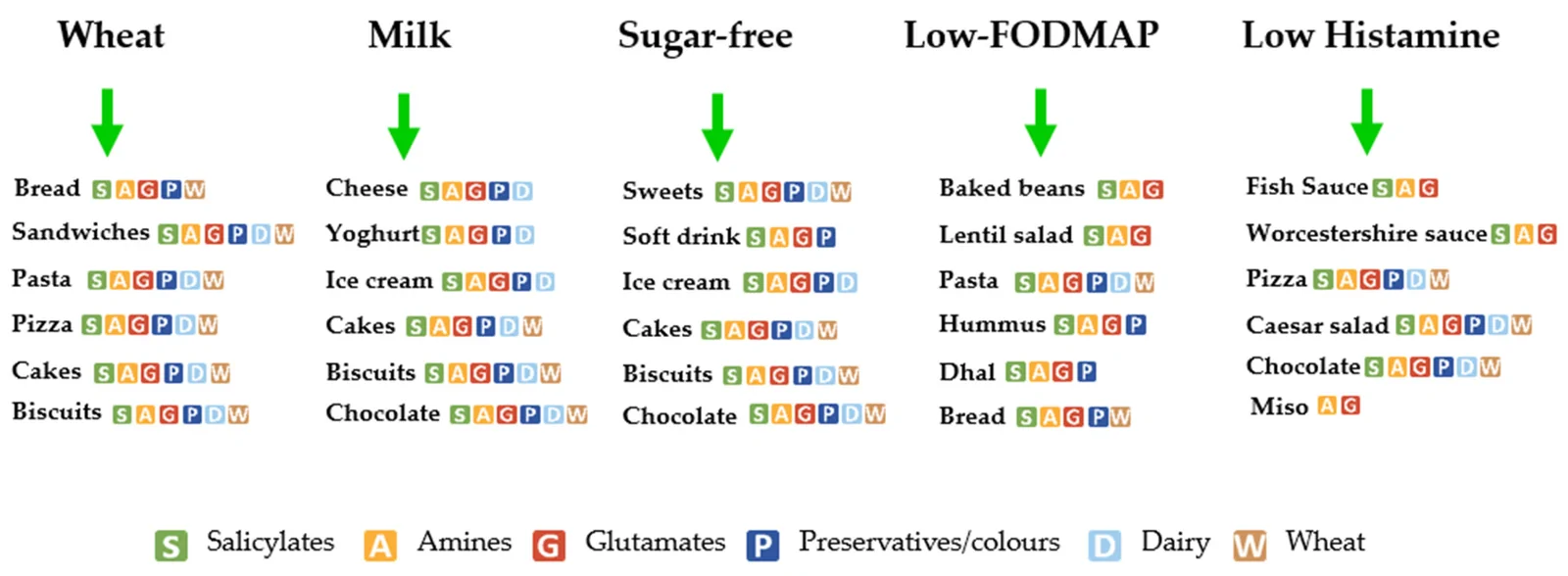
Popular dietary restrictions.

Figure 1 below shows some popular diets that when followed may concurrently reduce the intake of specific foods and the chemicals they contain. This includes salicylates, biogenic amines, and glutamate [139].

### 3.3. Elimination Diet

Adults and parents of children, at their first appointment, elected to follow either the strict elimination diet (10,074) (71.4%), moderate approach (2760) (19.5%), or simple approach (1285) (9.1%) for 2–6 weeks. Of these, 6645 (47.1%) became asymptomatic (≥80% symptom improvement) within two to six weeks, 628 (4.4%) reported no improvement, and 6846 (48.5%) were lost to follow-up.

Table 5 below outlines the number of patients who either improved or did not improve on the diet and those who did not complete the protocol.

### 3.4. Challenge Results

The patients who became asymptomatic (n = 6645) were offered testing with DBPC capsule challenges and/or food challenges [39,110,111,213,263] (Appendix C, Appendix D, Appendix E and Appendix F). Responses were recorded in a daily food/symptom diary. Of these, 4963 returned for interpretation of their DBPC capsule and/or food chemical challenges; 2429 patients had done open food challenges; 3326 had completed the DBPC capsule challenge protocol; and 1830 completed both DBPC capsule and food challenges (Table 5).

Although individual reactivity was idiosyncratic, a highly reproducible pattern was evident across the entire group. McNemar’s test for correlated proportions with Bonferroni correction was used to compare each DBPC chemical challenge response with all the other compounds given so that any significant correlations between the chemical substances and placebos could be made. The proportion reacting to each of the active chemical challenges versus sucrose and starch placebos was highly significant (*p* < 0.001) (Table 6 and Appendix H). In patients who underwent all food and/or DBPC challenges, the mean number of positive reactions was two reactions per patient for standard food challenges, and three reactions per patient for DBPC challenges.

While no multivariable regression model was applied to adjust for potential confounders, patients had to first achieve a prespecified degree of clinical improvement where they had ≥80% improvement from baseline or where symptoms had cleared or subsided significantly for five consecutive days. This elimination period was essential to exclude residual symptoms or other confounders such as age, sex, comorbidities, concomitant medications, and dietary adherence.

The patient then served as their own control, with the response to each challenge compared directly with the response to placebo. As the challenge protocol may have involved a wide array of food chemicals, the number of statistical comparisons could have increased the probability of obtaining at least one apparently positive result by chance or other reasons. A Bonferroni correction was therefore added to the McNemar’s analysis to provide a more conservative assessment of whether an individual compound produced a response over placebo.

To determine whether there was a different pattern of reactivity in patients on the strict elimination diet versus those on the moderate or simple diet approaches, the patient population was divided into three subgroups and the results tabulated separately (Table 7 and Appendix I). No significant difference was detected in the mean reaction rates following the strict elimination diet, moderate approach, or simple approach.

## 4. Discussion

As outlined, this study began in 1977 to test the hypothesis that CSU/AO can be caused by adverse reactions to food additives and salicylates [135]. At the time, the inadequacy of available data on the occurrence of dietary salicylates led to the laboratory analysis of a wide range of commonly eaten foods [48]. This information greatly simplified the dietary investigation and management of patients with food intolerance [39,110].

Once the importance of natural salicylates was recognized, it became easier to identify other compounds, found in apparently unrelated foods, capable of provoking a similar range of clinical symptoms [39]. These observations made it possible to gain a broader understanding of the nature of non-immunological food reactions, as well as providing a useful conceptual framework within which the investigation and management of individual patients could be rationally planned.

### 4.1. Clinical Observations

The majority of patients presenting to the Clinic with recognized or unrecognized adverse food reactions were shown to be sensitive to a range of natural and artificial food chemicals. Most patients reacted idiosyncratically to several compounds when challenged double-blind, and these results generally correlated well with clinical food reactions. Symptoms could involve the skin, gastrointestinal tract, respiratory tract, or central nervous system, with each patient manifesting an individual pattern of target organ responsiveness, independent of the chemical compounds involved.

Acute reactions behaved in a dose-dependent fashion, and they could be followed by tachyphylaxis, i.e., a “refractory” period (lack of responsiveness to a subsequent challenge), lasting up to three days. Chronic or recurrent symptoms were often more insidious and appeared to depend on the cumulative dose ingested from a variety of food sources over several days, or over two to three weeks in some cases. Most of these phenomena have also been documented by other investigators, including dose dependence, tachyphylaxis, tolerance, withdrawal reactions, and lowering of the dose-threshold with abstinence (super-sensitivity) [24,118,119,120,121].

The clinical behavior of these reactions strongly suggests an underlying pharmacological mechanism with abrupt dietary restrictions often being followed by withdrawal symptoms, and a fall in the dose–response threshold [110]. The use of a pharmacological “paradigm” made it possible to resolve much of the conflicting information in the literature regarding reactions to natural and artificial chemicals in food, making it possible to clarify some of the myths and reality of common food intolerances. Furthermore, it provided clinicians with a valuable conceptual framework to guide diagnosis and assist with day-to-day dietary management [27].

**Figure A3:**
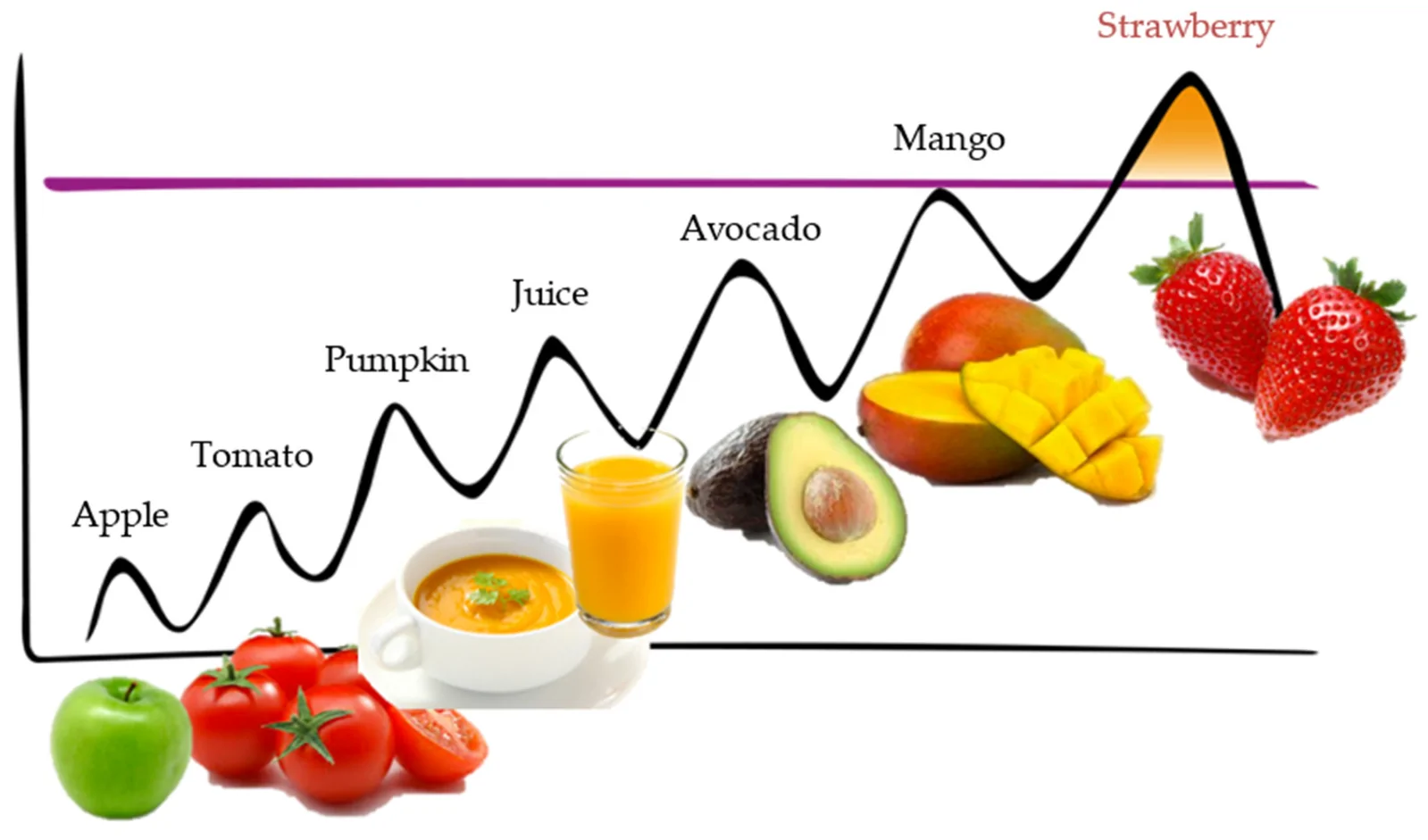
Cumulative effects of dietary salicylates.

**Figure A4:**
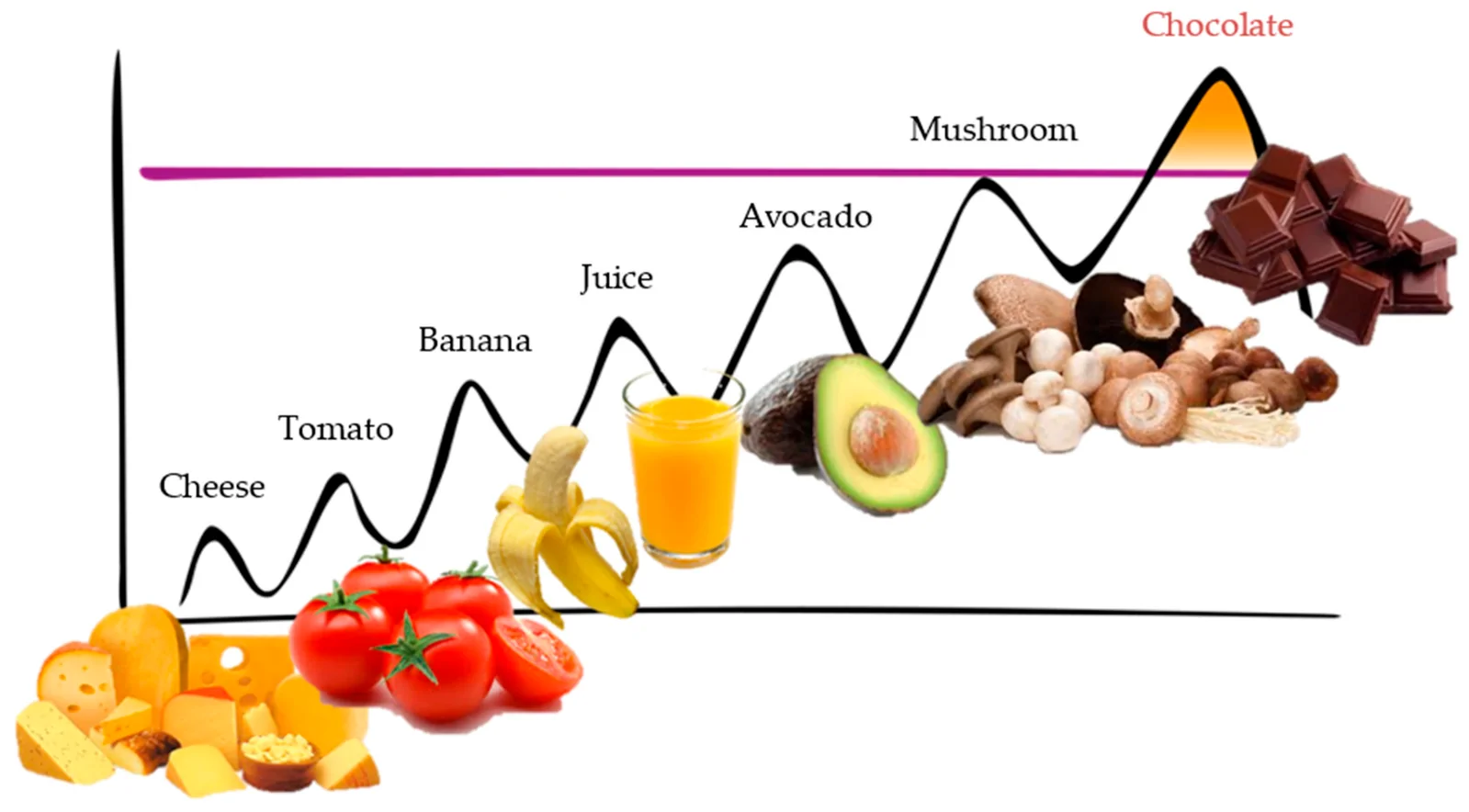
Cumulative effects of dietary biogenic amines.

Patients often reported that a particular food caused symptoms on some occasions but not others. The explanation became clear once it was appreciated that reactions depended on (a) the cumulative dose of the relevant chemicals from a variety of food sources, and (b) the patient’s dose-threshold, which may fluctuate depending on which foods had been eaten recently. A commonly encountered example of this was the amine-sensitive patient with migraine who had noticed that chocolate sometimes, but not always, triggered a headache. The diagrams shown in (Appendix J Figure A3 and Figure A4) were used to illustrate to such patients how a combination of food, eaten in varying amounts and at varying intervals, could determine whether or not a reaction might likely occur after a single large dose of salicylate or biogenic amines. Similarly, charts showing the salicylate, biogenic amine, and glutamate content of commonly eaten foods were developed and enabled sensitive patients to introduce more variety into their daily diet, at the same time, ensuring that they avoided the cumulative effects which could lead to a relapse of symptoms over several days or weeks [108,109,264].

Other popular dietary approaches, e.g., low-excitotoxin (low-glutamate) diet, low-FODMAP diet, low-gluten/wheat diet, low-histamine diet, pseudo-allergenic diet, and oligoantigenic diet, also eliminate a wide range of foods that are rich in natural chemicals, possibly explaining why people with food-chemical intolerances who follow these popular diets often feel better, at least temporarily. If health improves while on one of these diets, it is easy to jump to the conclusion that the problem has been identified. However, when symptoms recur (as they usually do), a more systematic approach to tracking down the offending chemicals needs to occur.

### 4.2. Interpretation of Challenge Results

Most of the challenge dosages were selected based on those used in previously published studies [128,129,131,133,134,135,136,138,207,208,210,211,212,214,215,216,217,242,243,244,245,246,247,248,249,250,251,253,254,255,256,257,258,259,260,261,271], taking into account the amounts likely to be consumed in a day on an average diet. The doses used for metabisulfite and MSG are two to three times those which might be consumed under normal circumstances, and that of aspirin is approximately 10–20 times the average daily intake. It could therefore be argued that the challenges represent an unnaturally large physiological exposure to these food substances, and that some healthy individuals may also react if tested. On the other hand, no single challenge given as a bolus dose can accurately reproduce the cumulative effects of long-term low-dose salicylate ingestion in the daily diet of a sensitive individual.

In the clinical context, the objective of challenge testing is to reliably identify the food substances responsible for provoking symptoms in patients selected because of a favorable response to dietary elimination. Ideally, normal control subjects should have been included in the present study in order to estimate the sensitivity, specificity, and predictive value of the challenge tests in the diagnosis of food intolerance [13,272,273]. However, the practical and ethical difficulties associated with placing normal individuals on a highly restricted diet for up to six weeks precluded this. Nevertheless, in the present study, challenge testing did appear to reliably identify the relevant foods symptomatic patients reacted to as judged by the follow-up results [110].

### 4.3. Possible Underlying Mechanisms

No single hypothesis has thus far satisfactorily explained the protean clinical manifestations of food intolerance, the relationship between aspirin and various other substances capable of provoking idiosyncratic reactions, and the associated pharmacological phenomena. However, an important clue may lie in the finding of a high incidence of reactivity to biogenic amines and MSG amongst our patients. These substances are not recognized as antimicrobials, inhibitors of prostaglandin production, or uncoupling agents in oxidative phosphorylation and are more likely to act via neurogenic mechanisms. The biogenic amines have well recognized neuro-humoral actions [274,275,276,277], and glutamate is an excitatory neurotransmitter in the central nervous system [276,277,278,279,280,281,282], suggesting the possibility of an underlying neuropharmacological abnormality involving regulatory neuro peptides [39].

This possible mechanistic hypothesis requires further validation by basic research, so that conclusions align with the scope of data support in this study.

### 4.4. Challenge and Limitations

This study enrolled only patients with suspected food intolerance from the Allergy Unit, and no healthy control group underwent the same elimination diet and challenge procedure. The findings primarily reflect response characteristics of the cohort.

The elimination phase of the diet does not establish causality. The strongest evidence for specific chemical triggers comes from the subsequent blinded challenge phase.

The elimination-diet period excluded residual symptoms and other potential confounders, such as age, sex, comorbidities, concomitant medications, and dietary adherence, so a multivariable regress model was not applied to adjust for potential confounders. This analysis method reduced the ability to attribute independent effects of each factor on challenge outcomes.

Table 5 reports that 48.49% of patients were technically lost to follow-up; however, the outcome for some of these patients is known. Given the magnitude of the loss to follow-up, this represents an important potential source of attrition/selection bias.

Strict adherence to the low-chemical diet requires a major alteration in eating patterns and this, together with the self-discipline needed during the two to three months of challenge, requires considerable motivation on the part of the patients [283,284].

N-of-1 studies in clinical research focus on the individual and are useful for evaluating individualized treatment responses, particularly within the context of personalized medicine and development of an effective personalized diet for long-term management and relief of symptoms [284].

While confidence intervals and effect size estimates quantify the extent and precision of a treatment effect, our challenge data was derived from multiple paired diagnostic challenge outcomes across numerous food chemicals administered within an N-of-1 framework rather than from a single intervention designed to estimate a common effect size. Participants did not all undergo identical challenge protocols, and the primary objective was to determine whether active challenges resulted in significantly more reactions than placebo within individuals. McNemar’s test for paired responses was therefore considered the most appropriate analytical approach. Given the very large sample size and the absence of a single underlying effect parameter, confidence intervals and conventional effect size measures would add limited interpretive value and would not materially alter the conclusions of this study.

At initial presentation, patients suspected of having food intolerance were given all the necessary instructions for dietary testing. Those whose symptoms improved on the elimination diet were asked to contact the dietitian and were sent a set of double-blind challenge capsules, with instructions to return for follow-up once these were completed. Overall, of 14,119 patients commenced on the elimination diet, 35.2% successfully completed the elimination and challenge protocol. The remainder were lost to follow-up, and it was originally assumed that most of these patients had experienced no improvement on the elimination diet.

However, when surveyed in 1988 and 1999, it was reported that a significant proportion of those who replied had restricted their diet, although they did not proceed with the challenges. Many of these patients self-modified their diet to manage their symptoms based on information and resources received [110,285].

The most common reasons for failure to complete the protocol related to the stringency and inconvenience of the elimination diet; however, many of these patients had arrived at a modified diet by trial and error, based on the information received from the Allergy Unit.

We therefore conclude that the prevalence of food intolerance amongst the patients is likely to be a conservative estimate, based on the proportion completing the elimination diet and challenge program.

The finding that the demanding nature of the elimination diet and challenge protocol was such that nearly half the patients presenting dropped out might be taken to indicate that this is an unsatisfactory means of dietary investigation. Notably, however, it was found that those who were most highly motivated to comply with the strictest diet were patients with the most disabling and chronic symptoms where the diet resulted in substantial improvement [110,285].

A stringent initial diet can therefore be regarded as a useful means of screening out those patients with relatively mild symptoms who would be unlikely to comply with a restricted diet in the long term, even if some of their symptoms were found to be food related.

Further research involving a follow-up questionnaire of the cohort from 2000 to 2024 to further assess the long-term dietary relevance and impact of the DEDCP is warranted.

## 5. Conclusions

It is often asserted that ensuring a healthy and balanced diet, with adequate fruit, vegetable, fiber, and fluid intake, is the most important objective for the dietary management of patients. However, for many patients, this approach provides little or no relief of symptoms, and sometimes can result in exacerbation of symptoms. The symptoms can have debilitating effects on the quality of life for individuals—personally, socially, and financially [4]. The cost to society is also significant with loss of work/school days and recourse to an already over-burdened healthcare system [6].

The diagnosis and management of patients with adverse food reactions can be complex due to the variety of presenting symptoms and offending compounds in food. It is important in managing patients to undertake a wholistic and thorough diagnosis of the presenting syndromes and symptoms. This is due to patients presenting with a variety of symptoms and triggers rather than a single trigger. If only a single trigger is addressed, it may result in unsuccessful patient management.

Much remains to be learned about the clinical features, as well as the underlying mechanisms, of food intolerance. The major findings of this study were recognition of the widespread distribution of natural salicylates, biogenic amines, and glutamate in common foods; the clinical spectrum of adverse reactions; and the idiosyncratic cross-reactivity between a wide range of natural and artificial substances in the diet. These findings led to the successful development of a systematic and reliable method for the routine investigation and management of food-sensitive patients, suitable for use in outpatient clinics or private practice.

In Australia, partly as a result of the present study, there is more general appreciation of the role of diet in CSU/AO, DGBI, and symptoms of the central nervous system and respiratory tract, in particular, the importance of naturally occurring compounds, as well as additives [101,286,287,288,289,290,291,292,293,294,295,296,297,298,299,300,301,302,303,304,305,306,307,308,309,310,311]. The DEDCP and other practical resources [108,109,264] developed by the Clinic are resources that can be used by health practitioners to successfully investigate and manage patients who present with complex adverse food reactions.

In the 48 years since the DEDCP was first introduced at RPAH, the protocol has been requested by numerous general practitioners, specialists, and dietitians throughout Australia. Since 1991, over 160,000 copies of *Friendly Food—The Essential Guide to Managing Common Allergies and Intolerances* [264] have been purchased worldwide in 37 countries, including the United States, New Zealand, United Kingdom, Canada, Germany, Ireland, France, and Holland. From 2017, over 20,000 self-published sets of *Food Intolerance Handbooks Volume 1—Diagnostic Elimination Diet* [108] and *Volume 2—Challenges, Liberalizing, And Living with Intolerances* [109] have been sold worldwide. These resources have had a local and international impact and meet the wide-ranging demand for evidence-based management of patients with food intolerance.

## Figures and Tables

**Table 1:** Special diets for specific disorders.

Special Diets	Disorders	References
Low-excitotoxin (low-glutamate) diet	Fibromyalgia	[145,146,147,148,149]
Low-FODMAP diet	Irritable bowel syndrome (IBS)	[150,151,152,153,154,155]
Low-gluten/wheat diet	Non-celiac gluten/wheat intolerance	[87,88,89,90,92,93,94,156,157,158,159]
Low-histamine diet	Migraine	[160,161,162,163,164,165,166]
	Urticaria, angioedema	[167,168,169,170,171]
	Mast cell activation syndrome (MCAS)	[172,173]
	Disorders of gut–brain interaction (DGBI)	[65,66,117,122,174,175,176,177,178,179]
Pseudo-allergenic diet	Urticaria	[115,170,180,181,182,183,184,185,186,187,188]
Oligoantigenic diet	Attention-deficit/hyperactivity disorder (ADHD)	[102,105,189,190,191,192,193,194]

**Table 2:** Presenting symptoms and clinical history symptoms.

	**At Presentation**						**Clinical History**					
	**All Patients**		**Cardio-Vascular**		**Genito-Urinary**		**All Patients**		**Cardio-Vascular**		**Genito-Urinary**	
	**N**	**%**	**N**	**%**	**N**	**%**	**N**	**%**	**N**	**%**	**N**	**%**
SKIN	3885	27.52	7	0.05	6	0.04	1315	9.31	26	0.18	20	0.14
GIT	2838	20.10	9	0.06	39	0.28	784	5.55	15	0.11	26	0.18
CNS	1448	10.26	28	0.20	18	0.13	295	2.09	13	0.09	11	0.08
RESP	853	6.04	3	0.02	1	0.01	325	2.30	7	0.05	2	0.01
OTHER	12	0.08			12	0.08						
CNS and GIT	1348	9.55	36	0.25	45	0.32	1316	9.32	43	0.30	132	0.93
SKIN and GIT	579	4.10	3	0.02	11	0.08	647	4.58	36	0.25	28	0.20
RESP and SKIN	551	3.90	5	0.04	2	0.01	761	5.39	32	0.23	14	0.10
RESP and GIT	323	2.29	7	0.05	3	0.02	450	3.19	23	0.16	28	0.20
CNS and RESP	323	2.29	7	0.05	1	0.01	282	2.00	13	0.09	15	0.11
CNS and SKIN	275	1.95	8	0.06	4	0.03	368	2.61	22	0.16	19	0.13
CNS, SKIN, and GIT	439	3.11	21	0.15	12	0.08	1240	8.78	77	0.55	140	0.99
CNS, RESP, and GIT	418	2.96	25	0.18	20	0.14	1820	12.89	121	0.86	250	1.77
RESP, SKIN, and GIT	318	2.25	16	0.11			810	5.74	52	0.37	49	0.35
CNS, RESP, and SKIN	182	1.29	13	0.09	3	0.02	551	3.90	29	0.21	40	0.28
CNS, RESP, SKIN, and GIT	327	2.32	33	0.23	11	0.08	3155	22.35	321	2.27	561	3.97
**Total**	**14,119**	**100.00**	**221**	**1.57**	**188**	**1.33**	**14,119**	**100.00**	**830**	**5.88**	**1335**	**9.46**
	**At Presentation**						**Clinical History**					
	**Patients Challenged**		**Cardio-Vascular**		**Genitourinary**		**Patients Challenged**		**Cardio-Vascular**		**Genitourinary**	
	**N**	**%**	**N**	**%**	**N**	**%**	**N**	**%**	**N**	**%**	**N**	**%**
SKIN	1217	24.52	2	0.04	0	0.00	429	8.64	8	0.16	9	0.18
GIT	1002	20.19	2	0.04	12	0.24	273	5.50	8	0.16	8	0.16
CNS	545	10.98	15	0.30	10	0.20	91	1.83	2	0.04	3	0.06
RESP	236	4.76	1	0.02	0	0.00	72	1.45	1	0.02		
OTHER	2	0.04			2	0.04						
CNS and GIT	553	11.14	15	0.30	12	0.24	480	9.67	21	0.42	48	0.97
SKIN and GIT	206	4.15	1	0.02	5	0.10	196	3.95	12	0.24	12	0.24
RESP and SKIN	196	3.95	4	0.08	1	0.02	266	5.36	14	0.28	5	0.10
RESP and GIT	116	2.34	1	0.02	1	0.02	133	2.68	6	0.12	10	0.20
CNS and RESP	121	2.44	2	0.04	1	0.02	95	1.91	3	0.06	5	0.10
CNS and SKIN	101	2.04	4	0.08	1	0.02	94	1.89	2	0.04	8	0.16
CNS, SKIN, and GIT	188	3.79	9	0.18	6	0.12	469	9.45	31	0.62	51	1.03
CNS, RESP, and GIT	175	3.53	9	0.18	9	0.18	723	14.57	46	0.93	96	1.93
RESP, SKIN, and GIT	108	2.18	4	0.08			273	5.50	20	0.40	15	0.30
CNS, RESP, and SKIN	70	1.41	7	0.14	3	0.06	193	3.89	11	0.22	17	0.34
CNS, RESP, SKIN, and GIT	127	2.56	12	0.24	7	0.14	1176	23.70	123	2.48	239	4.82
**Total**	**4963**	**100.00**	**88**	**1.77**	**70**	**1.41**	**4963**	**100.00**	**308**	**6.21**	**526**	**10.60**

**Table 3:** Common presentation diets.

Diets at Presentation	Total Patients (N = 14,119)	Total Patients (%)
**Regular/balanced diet**	9747	69.03
**Modified diets**	4372	30.97
Complementary, alternative, integrative health diets	2004	14.19
Self-modified diet	854	6.05
Elimination diet other	548	3.88
Plant-based diet	511	3.62
Low-FODMAP diet	281	1.99
Weight-management diet	234	1.66
Elimination diet RPAH (strict, moderate, simple, and FF)	224	1.59
Anti-inflammatory diet—detox/cleansing diet	184	1.30
Low-fat—saturated-fat/cholesterol diet	102	0.72
Plain/bland-food diet	84	0.59
Diabetic/low-GI diet	47	0.33
High-fiber diet	45	0.32
Fasting	37	0.26
Food family/rotation diet	32	0.23
Low-salt diet	27	0.19
Organic food diet	24	0.17
Paleolithic diet	23	0.16
Low-fiber diet	18	0.13
Hypoglycemia diet	17	0.12
Celiac-disease gluten-free diet	14	0.10
Kosher/halal diet	14	0.10
Other diets *	12	0.08
Allergy diet	11	0.08

* Low-nickel diet, high-salt diet, low-alkaline diet, low-goitrogenic foods diet, low-L-arginine diet, low-oxalate diet, low-potassium diet, low-protein diet, low-tannin diet, low-vitamin-K diet, and renal diet.

**Table 4:** Common provoking food and other triggers.

Food Triggers	Chemical Content *	Total Patients (N = 14,119)	Total Patients (%)
Dairy foods	(S, A, G, P, C)	4157	29.44
Drinks, tea, coffee, and alcohol	S, A, G, (P, C)	4008	28.39
Meals/restaurant/takeaway	S, A, G, (P, C)	3733	26.44
Fruit	S, A, G, (P)	3576	25.33
Gluten-containing foods	(S, A, G, P, C)	3477	24.63
Vegetables	(S, A, G, P, C)	2956	20.94
Sugary foods and sweets	(S, A, G, P, C)	2453	17.37
Herbs and spices	S	1562	11.06
Meats	(S, A, G, P, C)	1294	9.16
Seafoods	(S, A, G, P, C)	1103	7.81
Sauces and dressings	(S, A, G, P, C)	927	6.57
Nuts and seeds	S, A	864	6.12
Eggs	(S, A, G, P, C)	719	5.09
MSG	G	619	4.38
Preservatives and antioxidants	P	573	4.06
Yeast and yeast extracts	S, A, G	478	3.39
Soy	(S, A, G, P)	473	3.35
Legumes	(S, A, G)	464	3.29
Salicylates	S	348	2.46
Artificial colors	C	345	2.44
Gluten-free foods	(S, A, G, P, C)	328	2.32
Formula/breast milk		315	2.23
Fats and oils	(S, A, G, P, C)	282	2.00
Flavors	S, A, G	162	1.15
Acidic foods	S	154	1.09
Amines	A	149	1.06
Coconut	S, A, (P, C)	119	0.84
Pickled foods	S, A, G, (P, C)	111	0.79
Additives	P, C	110	0.78
Condiments	S, A, G, (P, C)	106	0.75
**Other Triggers**			
Other, e.g., stress, exercise, and weather		4286	30.36
Medications		2740	19.41
Personal and home environment		2617	18.54
Toiletries and personal care		805	5.70
Nutritional supplements		310	2.20
**No Triggers**		2497	17.69

* S = Salicylate, A = biogenic amines, G = glutamate, P = preservative, C = color.

**Table 5:** Dietary results of patients on the elimination diet.

Number of Patients		Strict Elimination Diet (n = 10,074)	%	Moderate Elimination Diet (n = 2760)	%	Simple Elimination Diet (n = 1285)	%	All Elimination Diets (n = 14,119)	%
**Improvement**	**All**	**4935**	**48.99**	**1415**	**51.27**	**295**	**22.96**	**6645**	**47.06**
	Female	3627	36.00	1003	36.34	199	15.49	4829	34.20
	Male	1308	12.98	412	14.93	96	7.47	1816	12.86
**No improvement**	**All**	**493**	**4.89**	**107**	**3.88**	**28**	**2.18**	**628**	**4.45**
	Female	329	3.27	77	2.79	20	1.56	426	3.02
	Male	164	1.63	30	1.09	8	0.62	202	1.43
**Lost to follow-up**	**All**	**4646**	**46.12**	**1238**	**44.86**	**962**	**74.86**	**6846**	**48.49**
	Female	3325	33.01	884	32.03	663	51.60	4872	34.51
	Male	1321	13.11	354	12.83	299	23.27	1974	13.98
**Improvement and challenged**	**All challenged**	**3782**	**37.54**	**1066**	**38.62**	**115**	**8.95**	**4963**	**35.15**
	DBPC challenges	661	6.56	543	19.67	22	1.71	1226	8.68
	DBPC and food challenges	1815	18.02	277	10.04	38	2.96	2130	15.09
	Food challenges	1306	12.96	246	8.91	55	4.28	1607	11.38

**Table 6:** Challenge responses—all elimination diets (strict, moderate, and simple).

Compounds	DBPC		DBPC and Food		Food		All Challenges	
	Patients (n = 3326)	% Positive	Patients (n = 1830)	% Positive	Patients (n = 2429)	% Positive	Patients (n = 4963)	% Positive
**Amines**								
Amines	1599	47.22	621	65.86	1197	61.24	3417	55.52
**Antioxidant**								
Antioxidant	2120	39.25	70	71.43	373	40.75	2563	40.34
**Baker’s yeast**								
Baker’s yeast	254	35.43					254	35.43
**Brewer’s yeast**								
Brewer’s yeast	1368	35.89					1368	35.89
**Colors**								
Annatto	76	47.37					76	47.37
Beta-carotene	559	15.38					559	15.38
Beta-carotene and annatto	116	42.24					116	42.24
Colors					493	46.86	493	46.86
Erythrosine	435	40.69					435	40.69
Green—tartrazine and brilliant Blue	869	42.46					869	42.46
Orange—tartrazine and erythrosine	336	38.10					336	38.10
Tartrazine	1746	39.52					1746	39.52
**Glutamate**								
Glutamate	2253	53.93	111	55.86	468	56.84	2832	54.48
**Gluten and wheat**								
Gluten	1990	23.87					1990	23.87
Wheat					2400	52.92	2400	52.92
**Green cordial**								
Green cordial	133	34.59					133	34.59
**Milk and lactose**								
Lactose	2374	21.61					2374	21.61
Milk					2352	52.08	2352	52.08
Lactose-free milk					209	53.11	209	53.11
**Nitrate**								
Nitrate	2152	42.80	62	61.29	259	36.29	2473	42.58
**Preservatives**								
Benzoate	659	50.23			272	45.96	931	48.98
Benzoates/sorbate	419	42.24			10	50.00	429	42.42
Benzoates/sorbate/sulfite	1711	48.80	20	70.00	57	56.14	1788	49.27
Benzoates/sulfite	66	54.55					66	54.55
Sorbate	67	47.76			202	36.63	269	39.41
Sulfite	1008	49.40			250	56.80	1258	50.87
**Propionate**								
Propionate	1760	41.53	72	59.72	364	40.93	2196	42.03
**Salicylate**								
Aspirin	2844	51.44					2844	51.44
Salicylate					2057	62.23	2057	62.23
Sodium salicylate	463	43.84					463	43.84
**Soy**								
Soy					337	61.13	337	61.13
**Starch**								
Starch	1453	19.20					1453	19.20
**Sucrose**								
Sucrose (1)	1796	16.31					1796	16.31
Sucrose (2)	540	21.85					540	21.85

**Table 7:** Challenge responses—strict, moderate, or simple elimination diet.

Compounds	Strict Elimination Diet		Moderate Elimination Diet		Simple Elimination Diet		All Elimination Diets	
	Patients (n = 3782)	% Positive	Patients (n = 1066)	% Positive	Patients (n = 115)	% Positive	Patients (n = 4963)	% Positive
**Amines**								
Amines	2822	55.4	518	55.98	77	55.84	3417	55.52
**Antioxidant**								
Antioxidant	2142	40.9	368	37.23	53	37.74	2563	40.34
**Baker’s yeast**								
Baker’s yeast	120	35.00	132	36.36	2	0.00	254	35.43
**Brewer’s yeast**								
Brewer’s yeast	782	36.83	576	34.72	10	30.00	1368	35.89
**Colors**								
Annatto	59	47.46	16	50.00	1	0.00	76	47.37
Beta-carotene	240	31.25	314	3.50	5	0.00	559	15.38
Beta-carotene and annatto	11	54.55	104	41.35	1	0.00	116	42.24
Colors	399	47.62	77	45.45	17	35.29	493	46.86
Erythrosine	421	40.14	14	57.14			435	40.69
Green—tartrazine and brilliant blue	728	42.72	119	39.50	22	50.00	869	42.46
Orange—tartrazine and erythrosine	322	38.51	8	12.50	6	50.00	336	38.10
Tartrazine	1119	39.68	608	38.98	19	47.37	1746	39.52
**Glutamate**								
Glutamate	2364	53.81	405	58.02	63	57.14	2832	54.48
**Gluten and wheat**								
Gluten	1685	24.45	272	20.96	33	18.18	1990	23.87
Wheat	2077	52.58	279	56.99	44	43.18	2400	52.92
**Green cordial**								
Green cordial	128	32.81	5	80.00			133	34.59
**Milk and lactose**								
Lactose	1659	23.45	680	16.76	35	28.57	2374	21.61
Milk	2045	51.83	268	54.85	39	46.15	2352	52.08
Lactose-free milk	154	52.60	47	57.45	8	37.50	209	53.11
**Nitrate**								
Nitrate	2072	41.51	355	49.58	46	36.96	2473	42.58
**Preservatives**								
Benzoate	433	50.58	483	47.41	15	53.33	931	48.98
Benzoates/sorbate	370	42.97	48	35.42	11	54.55	429	42.42
Benzoates/sorbate/sulfite	1496	48.06	259	57.53	33	39.39	1788	49.27
Benzoates/sulfite	54	50.00	11	72.73	1	100.00	66	54.55
Sorbate	194	42.27	66	31.82	9	33.33	269	39.41
Sulfite	802	54.61	436	43.81	20	55.00	1258	50.87
**Propionate**								
Propionate	1927	42.81	219	38.36	50	28.00	2196	42.03
**Salicylate**								
Aspirin	2073	49.06	722	58.03	49	55.10	2844	51.44
Salicylate	1677	63.09	318	57.86	62	61.29	2057	62.23
Sodium salicylate	147	38.78	311	45.66	5	80.00	463	43.84
**Soy**								
Soy	280	57.86	46	78.26	11	72.73	337	61.13
**Starch**								
Starch	1254	19.94	178	13.48	21	23.81	1453	19.20
**Sucrose**								
Sucrose (1)	1529	16.87	244	13.93	23	4.35	1796	16.31
Sucrose (2)	431	22.27	91	20.88	18	16.67	540	21.85

**Figure A1:**
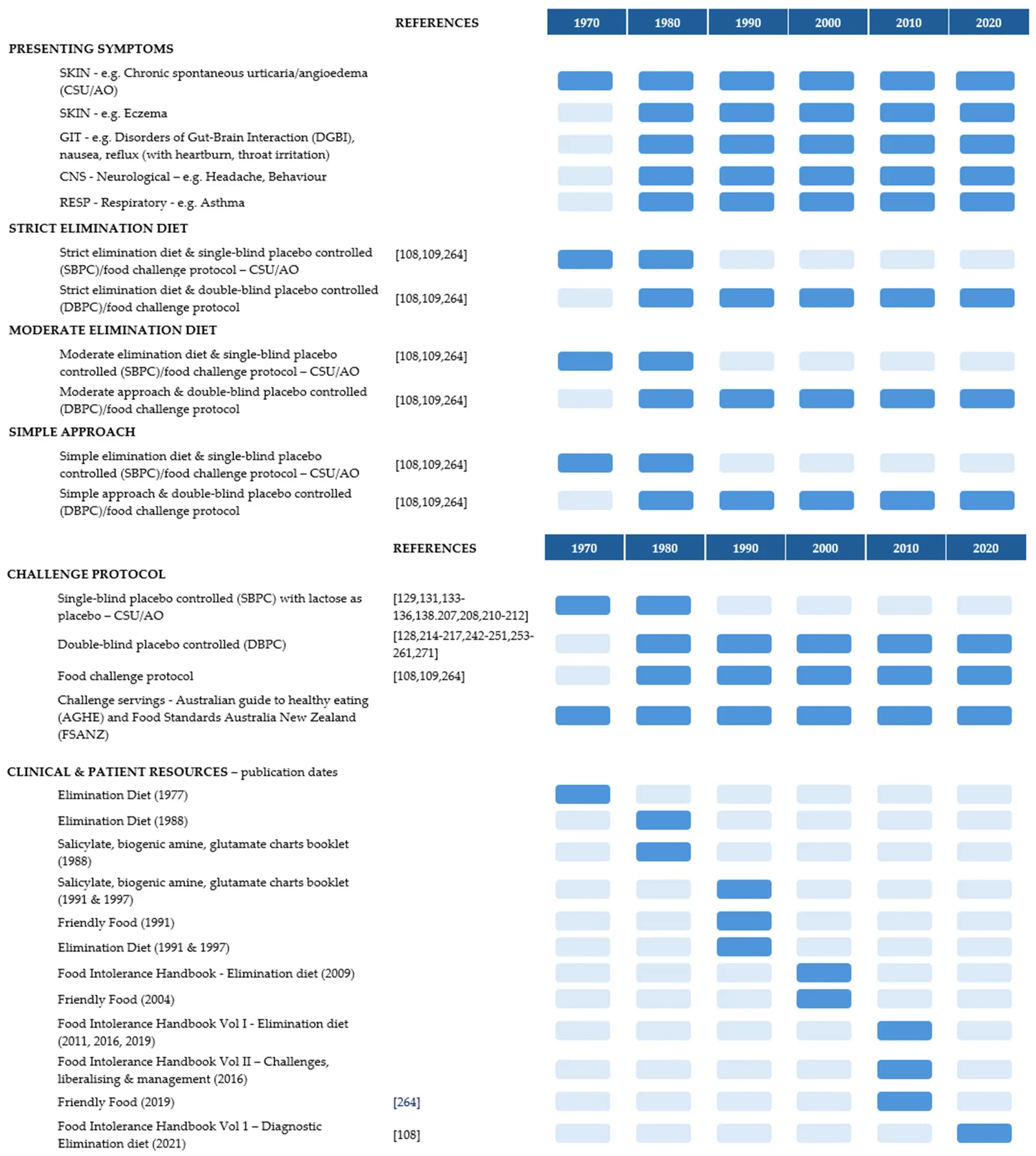

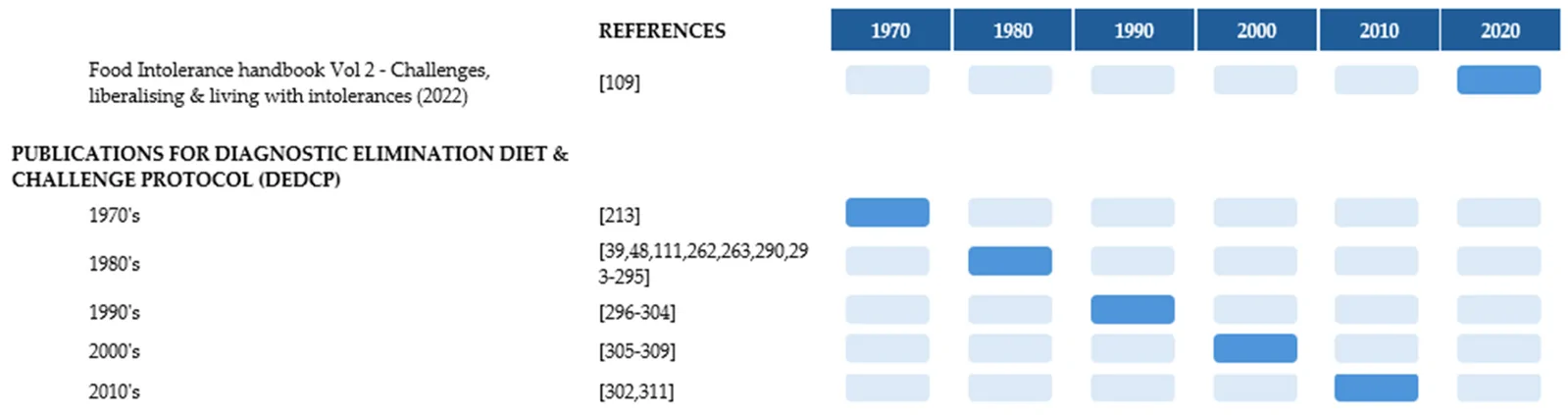
Development of the Diagnostic Elimination Diet and Challenge Protocol.

**Figure A2:**
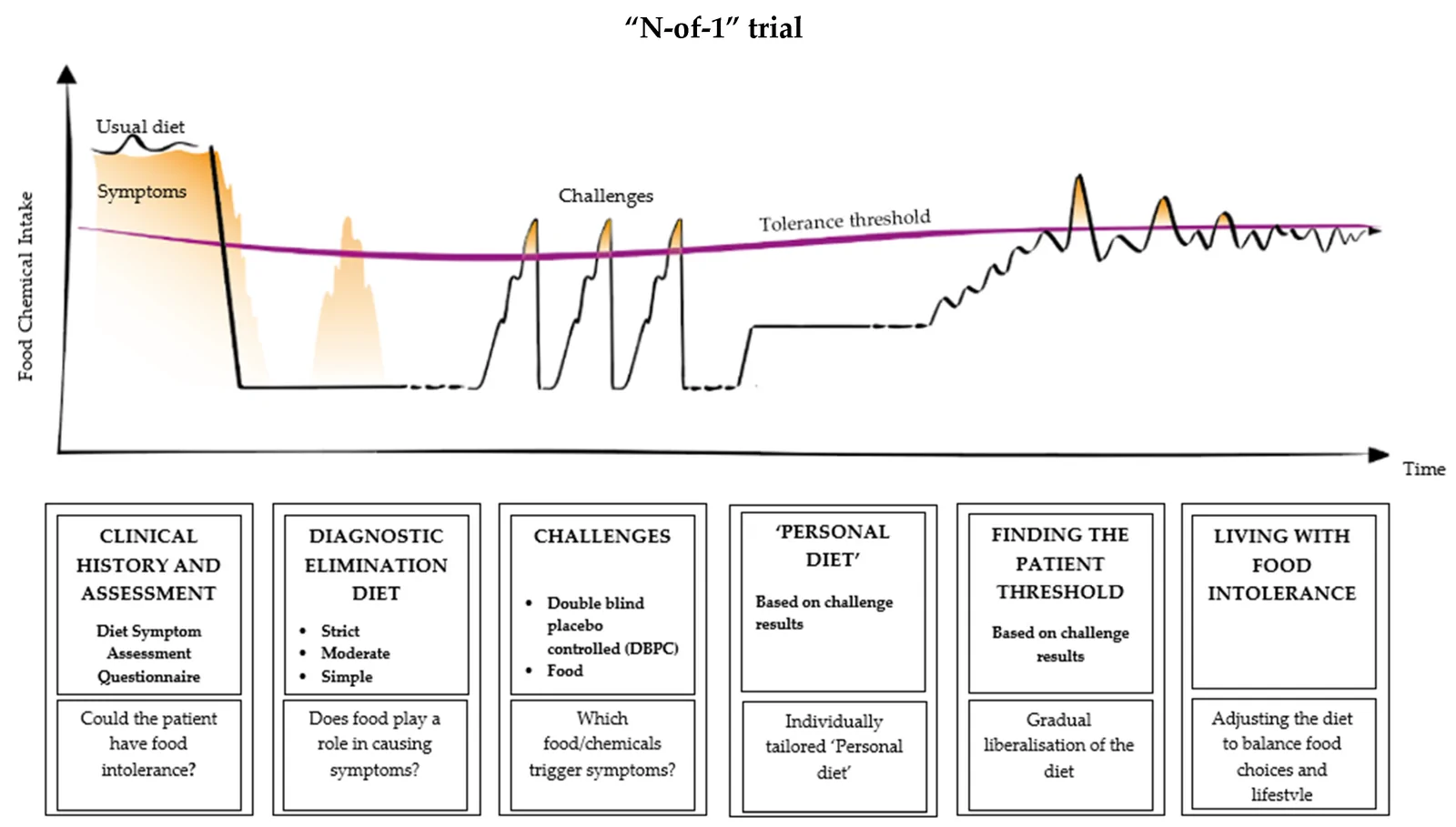
Diagnostic Elimination Diet and Challenge Protocol.

## Data Availability

The relevant data is stored in a bespoke RPAH Allergy Clinic database that securely maintains clinical patient records and associated study data. The database is hosted on a secure SQL NSW Health Azure Cloud server and is protected by strict access controls, with access granted only to authorized members of the Allergy Unit IT team. The database was queried in real time, and analyses were performed directly within the security system. This ensured data remained always contained within the secure environment. There were no publicly archived datasets analyzed or generated during this study, due to privacy and ethical restrictions. This article outlines the research data that can be made public.

## Abbreviations

The following abbreviations are used in this manuscript:

ADHD	Attention-deficit/hyperactivity disorder
AGHE	Australian Guide to Healthy Eating
ARFID	Avoidant/restrictive food intake
ASD	Autistic spectrum disorder
CD	Celiac disease
CNS	Central nervous system
CSU/AO	Chronic Spontaneous Urticaria and Angioedema
DBPC	Double-blind, placebo-controlled
DEDCP	Diagnostic Elimination Diet and Challenge Protocol
DGBIs	Disorders of gut–brain interaction
DHQ	Diet History Questionnaire
DSAQ	Diet Symptom Assessment Questionnaire
EoE	Eosinophilic Esophagitis
FPIES	Food protein-induced enterocolitis syndrome
FI	Food intolerance
FSANZ	Food Standards Australia New Zealand
FODMAPs	Fermentable Oligosaccharides, Disaccharides, Monosaccharides, and Polyols
GIT	Gastrointestinal tract
HPLC	High-performance liquid chromatography
IBD	Inflammatory bowel disease
IBS	Irritable bowel syndrome
MSG	Monosodium glutamate
PMT	Premenstrual tension
RESP	Respiratory tract
RPAH	Royal Prince Alfred Hospital
SKIN	Skin symptoms
SBPC	Single-blind, placebo-controlled
TLC	Thin-layer chromatography

## Appendix C

**Table A1:** Standard set and short set capsule challenges—double-blind, placebo-controlled (DBPC) (June 1997–December 2024).

Compound	Capsule	Standard Set (≥18 Years Old)	Short Set (Adults Only)	Childrens Set (8–18 Years Old)	E Code	Food Standards Australia New Zealand (FSANZ)	Food Equivalent
	A and B	Dose	Dose	Dose			
Acetylsalicylic acid (aspirin) **	A	600 mg		300 mg			
	B	600 mg		300 mg			
Tyramine hydrochloride		150 mg	150 mg	150 mg			120 g cheese
ß-Phenethylamine hydrochloride		4 mg	4 mg	4 mg			120 g chocolate
Monosodium glutamate (MSG) **	A	2.5 g		1.25 g	621		
	B	2.5 g		1.25 g	621		
Sodium benzoate	A	500 mg	500 mg	500 mg	211	400 mg/kg	1200 mL lemonade
4 Hydroxy benzoic acid	A	200 mg	200 mg	200 mg	214	400 mg/kg	500 mL lemonade
Sorbic acid	A	200 mg	200 mg	200 mg	200	3 g/kg	70 g cottage cheese
Sodium metabisulfite **	B	500 mg		250 mg	223	300 mg/kg	1 kg dehydrated potato or 666 g dehydrated French beans
Calcium propionate		500 mg	500 mg	500 mg	282	2.4 g/kg	208 g of bread (10 thin slices)
Sodium nitrate		25 mg	25 mg	12.5 mg	251	500 mg/kg	50 g meat
Sodium nitrite		25 mg	25 mg	12.5 mg	250	125 mg/kg	200 g meat
Butylated hydroxy anisole		50 mg	50 mg	50 mg	320	200 mg/kg	250 mL oil = 550 g French fries or 650 g crisps
Butylated hydroxy toluene		50 mg	50 mg	50 mg	321	200 mg/kg	250 mL oil = 550 g French fries or 650 g crisps
Tartrazine		30 mg	30 mg	30 mg	102	30 mg/kg in drinks or 300 mg/kg in sweets	
Brilliant blue		10 mg	10 mg	10 mg	133	30 mg/kg in drinks or 300 mg/kg in sweets	
Gluten		1.5 g	1.5 g	1.5 g			½–1 slice of bread
Lactose		0.5 g	0.5 g	0.5 g			2 teaspoons milk
Starch		1 g	1 g	1 g			
Sucrose		1 g	1 g	1 g			
Sucrose	A	1 g	1 g	1 g			
	B	1 g	1 g	1 g			

Graded dose challenges are labeled A and B. If there is no reaction after 2 h, the B dose is taken. ** ADULTS with a history of ASTHMA, TONGUE OR THROAT SWELLING, OR ANAPHYLAXIS will need to take these challenges in HOSPITAL under MEDICAL SUPERVISION—SUPERVISED CAPSULE CHALLENGES SET. NOTE: CHILDREN with asthma to ONLY do FOOD challenges for aspirin, sodium metabisulfite and monosodium glutamate [128,129,131,133,134,135,136,138,207,208,210,211,212,214,215,216,217,242,243,244,245,246,247,248,249,250,251,253,254,255,256,257,258,259,260,261,271].

## Appendix D

**Table A2:** Supervised capsule challenges set—taken under medical supervision (June 1997–December 2024).

Supervised Capsule Challenges Set **		
500 mg Sodium metabisulfite in 4 doses (50, 100, 150, 200 mg doses). Note: Also has 500 mg citric acid/dose.		
5 gm Monosodium glutamate (MSG) in 3 doses (1 gm, 1.5 gm, 2.5 gm)		
1.2 g Aspirin challenged in 5 doses (50, 100, 150, 300, 600 mg doses).		
Supervised Capsule Challenges Set **		
** ADULTS ONLY—with asthma, laryngeal edema—Challenges MUST be taken under medical supervision		
Compound	Dose	Total Dose
Acetylsalicylic acid (aspirin)	1	50 mg
Acetylsalicylic acid (aspirin)	2	100 mg
Acetylsalicylic acid (aspirin)	3	150 mg
Acetylsalicylic acid (aspirin)	4	300 mg
Acetylsalicylic acid (aspirin)	5	600 mg
Monosodium glutamate	1	1 g
Monosodium glutamate	2	1.5 g
Monosodium glutamate	3	2.5 g
Sodium metabisulfite *	1	50 mg *
Sodium metabisulfite *	2	100 mg *
Sodium metabisulfite *	3	150 mg *
Sodium metabisulfite *	4	200 mg *

* Plus, 500 mg citric acid in 100 mL of water. ** NOTE: CHILDREN with asthma to ONLY do FOOD challenges for aspirin, sodium metabisulfite, and monosodium glutamate.

## Appendix E

**Table A3:** Capsule challenges’ development (April 1977–December 2024).

Compound		April ’77–Jan ’79	Jan ’79–Jan ’83	Jan ’82–Jan ’83	Jan ’83–Dec ’85		Nov ’81–May ’84	May ’84–Jan ’85	Jan ’85–May ’86	May ’86–May ’89	May ’89–June ’97	June ’97–Dec ’24
		CSU/AO #	CSU/AO #	CSU/AO #	CSU/AO #		FI *	FI *	FI # and CSU/AO #	FI * and CSU/AO #	FI * and CSU/AO #	FI * and CSU/AO #
	A and B	SBPC	SBPC	SBPC	DBPC	A and B	DBPC	DBPC	DBPC	DBPC	DBPC	DBPC
Acetylsalicylic acid (aspirin) **	A	150 mg	150 mg	150 mg	300 mg	A	600 mg	600 mg	600 mg	600 mg	600 mg	600 mg
	B	300 mg	300 mg	300 mg		B	600 mg	600 mg	600 mg	600 mg	600 mg	600 mg
Sodium salicylate	A		150	150	300 mg	A						
Sodium salicylate	B		300	300		B						
Tyramine hydrochloride				150 mg	150 mg		150 mg	150 mg	150 mg	150 mg	150 mg	150 mg
ß-Phenethylamine hydrochloride				4 mg	4 mg		4 mg	4 mg	4 mg	4 mg	4 mg	4 mg
Monosodium glutamate (MSG) **	A			2.5 g	2.5 g	A	2.5 g	2.5 g	2.5 g	2.5 g	2.5 g	2.5 g
	B			2.5 g	2.5 g	B	2.5 g	2.5 g	2.5 g	2.5 g	2.5 g	2.5 g
Sodium benzoate		500 mg	500 mg	500 mg	500 mg		500 mg	500 mg	500 mg	500 mg	500 mg	500 mg
4 Hydroxy benzoic acid		200 mg	200 mg	200 mg	200 mg		200 mg	200 mg	200 mg	200 mg	200 mg	200 mg
Sorbic acid				200 mg	200 mg		200 mg	200 mg	200 mg	200 mg	200 mg	200 mg
Sodium metabisulphite **			500 mg	500 mg	500 mg		500 mg	500 mg	500 mg	500 mg	500 mg	500 mg
Calcium propionate								500 mg	500 mg	500 mg	500 mg	500 mg
Sodium nitrate							100 mg	50 mg	25 mg	25 mg	25 mg	25 mg
Sodium nitrite							100 mg	50 mg	25 mg	25 mg	25 mg	25 mg
Butylated hydroxy anisole				50 mg	50 mg		50 mg	50 mg	50 mg	50 mg	50 mg	50 mg
Butylated hydroxy toluene				50 mg	50 mg		50 mg	50 mg	50 mg	50 mg	50 mg	50 mg
Tartrazine		10 mg	10 mg	30 mg	30 mg		30 mg	30 mg	30 mg	30 mg		
Green—tartrazine and brilliant blue								24 mg and 6 mg	24 mg and 6 mg			30 mg and 10 mg
Erythrosine								30 mg	30 mg	30 mg		
Orange—tartrazine and erythrosine											30 mg and 30 mg	
Annatto (+sucrose)							30 μL					
Green cordial—green—tartrazine and brilliant blue								24 mg and 6 mg	24 mg and 6 mg			
Green cordial—sodium benzoate and sodium metabisulphite **								80 mg and 23 mg	80 mg and 23 mg			
B carotene (+starch)				100 g and 600 mg	100 g and 600 mg		100 g and 600 mg					
B carotene (+starch) and Annatto (+sucrose)							100 mg and 30 μL					
Brewer’s yeast		600 mg		600 mg	600 mg		600 mg	600 mg	600 mg	600 mg		
Baker’s yeast (fresh)							1.5 g	1.5 g	1.5 g	1.5 g		
Baker’s yeast (dried)							1.5 g	1.5 g	1.5 g	1.5 g		
Gluten							1.5 g	1.5 g	1.5 g	1.5 g	1.5 g	1.5 g
Lactose		700 mg		700 mg	500 mg		500 mg	500 mg	500 mg	500 mg	500 mg	500 mg
Starch							1 g	1 g	1 g	1 g	1 g	1 g
Sucrose (1)							1 g	1 g	1 g	1 g	1 g	1 g
Sucrose (2)	A					A		1 g	1 g	1 g	1 g	1 g
	B					B		1 g	1 g	1 g	1 g	1 g

Graded dose challenges are labeled A and B. If there is no reaction after 2 h, the B * dose is taken; # CSU/AO—Chronic Spontaneous Urticaria/Angioedema; # FI—food-intolerance symptoms—skin, GIT, CNS, respiratory symptoms. ** ADULTS with a history of ASTHMA, TONGUE OR THROAT SWELLING, OR ANAPHYLAXIS will need to take these challenges in HOSPITAL under MEDICAL SUPERVISION—SUPERVISED CAPSULE CHALLENGES SET. NOTE: CHILDREN with asthma to ONLY do FOOD challenges for aspirin, sodium metabisulfite and monosodium glutamate [128,129,131,133,134,135,136,138,207,208,210,211,212,214,215,216,217,242,243,244,245,246,247,248,249,250,251,253,254,255,256,257,258,259,260,261,271].

Modifications to Challenge Set

- Sodium salicylate added for comparison with aspirin;
- Addition of sodium metabisulphite, a widely used preservative;
- Preservative challenge of combined sodium benzoate, 4 hydroxy benzoic acid, sorbic acid, and sodium metabisulphite as used in combination in food;
- Addition of propionate, a common preservative of yeast leavened bakery items in the 1980s;
- Reduced dose of nitrate as some patients experienced severe reactions;
- Increased dose of tartrazine from 10 mg to 30 mg which is equivalent to one food item;
- Several color challenges and a composite color and preservative challenge given so that correlations could be made;
- Addition of B carotene and omission because it was found to be contaminated—had strong smell of carrots;
- Deletion of baker’s yeast because it was found to be denatured by baking;
- Lactose placebo replaced with starch placebo and sucrose placebo.

## Appendix F

**Table A4:** Food Challenges (1980 and onwards).

Food Challenge	Foods	Challenge Time
Salicylate	Apple, apricot, blueberries, peach, rock melon, strawberries, watermelon	Up to 7 days
	Asparagus, beetroot, capsicum, carrot, cucumber, onion, pumpkin, sweet potato, zucchini	
	Herbs, spices	
	Apple juice (unpreserved), coffee, tea	
	Honey, peppermints (no color)	
Amine	Banana (ripe)	Up to 7 days
	Plain dark chocolate	
	Cacao or cocoa powder	
	Pawpaw	
	Pork, salmon, sardines, tuna	
	If dairy tolerated,	
	cheddar cheese (mild, tasty), Swiss cheese, drinking chocolate, milk chocolate	
Glutamate	Chicken salt	Up to 3 days
	If dairy tolerated, parmesan cheese	
	If amines tolerated, soy sauce, tamari	
	If salicylate tolerated, flavor sachet from instant noodles	
Antioxidant	Hot commercial French fries cooked in oil containing antioxidants (310–312, 319–321)	Up to 3 days
Food color	100s and 1000s of cake-decorating sprinkles (artificially colored)	Up to 3 days
	Artificial liquid food colors (102, 104, 110, 122, 123, 124, 129, 132, 133, 142, 143, 151, 155)	
Propionate	Crumpets, pita bread preserved with propionate (282)	Up to 3 days
Nitrate and Nitrite	Ham, bacon, corned beef with added nitrate or nitrite (249–252)	Up to 3 days
Sorbate	Commercial lemonade preserved with 202	Up to 3 days
	If dairy tolerated, cottage cheese or ricotta cheese with 202	
Benzoate	Commercial lemonade preserved with 211	Up to 3 days
Sulfite	Dried pears preserved with 220–228	Up to 3 days
Wheat	Wheaten pasta	Up to 7 days
	Whole wheat grain biscuits	
	Unpreserved white or whole meal bread or bread rolls	
Milk	Cow’s milk	Up to 7 days
Soy milk	Soy milk, unflavored	Up to 7 days

## Appendix G

**Table A5:** Syndromes and symptoms.

Syndromes and Symptoms			
**Skin**	Urticaria—acute	Angioedema—acute	Nappy rash/perianal rash
	Urticaria—episodic	Angioedema—episodic	Perioral rash
	Urticaria—recurrent	Angioedema—recurrent	Rashes on the trunk
	Urticaria—cold	Eczema	Rashes under neck or behind ears
	Urticaria—exercise/cholinergic	Flushing/rosacea	
	Urticaria—other physical	Itch (without rash)	
**Gastrointestinal (GIT)**	Dysphagia	Esophageal pain	Diarrhea—urgency/explosive
	Gagging	Reflux	Diarrhea—watery/runny
	Heartburn	Sensation of food sticking	Distension
	Impaction	Tongue-swelling/angioedema	Irritable bowel syndrome (IBS)
	Indigestion	Unwell/sick	Pain/colic/cramps
	Itchy mouth	Vomiting	Wind/gas
	Itchy throat	Bloating/discomfort	Faltering growth
	Mouth Ulcers	Constipation	Feeding problem
	Nausea	Diarrhea—loose stools	Food refusal/spitting out food
**Respiratory**	Asthma	Laryngeal—swelling/edema	Rhinitis/sinus
	Chest tightness	Nasal itch	Runny nose
	Short of breath	Night cough	Sneezing
	Wheezing	Pharynx—swelling/edema	Croup
	Choking	Throat irritation	Sleep apnea
	Cough	Polyps	Snoring
	Hoarse	Post-nasal drip	
**Central** **Nervous** **System (CNS)**	ADHD	Fatigue/lethargy	Migraine—nausea
	ASD	Headache	Migraine—pain (abdominal)
	Chronic fatigue syndrome	Hyperactive/restless	Migraine—photophobia
	Fibromyalgia	Irritable	Migraine—vision
	Aches and pains/myalgia	Learning difficulties	Migraine—vomiting
	Aggressive/violent	Lightheaded/feeling faint	Panic attacks
	Anxiety	Loss of consciousness	Paresthesia/tingling/numbness
	Blurred vision	Malaise	Poor concentration/cognitive
	Collapse/floppy	Migraine	Sleep disturbance
	Depression	Migraine—aura	Tinnitus
	Difficult (oppositional)	Migraine—diarrhea	
	Dizziness	Migraine—dizziness	
**Genitourinary**	Bladder irritation	Dysmenorrhea	Vaginal discharge/irritation
	Burning urine	PMT/hormonal changes	Vulvodynia
	Frequent urination	Polycystic ovarian syndrome	
	Endometriosis	Vaginal thrush	
**Cardiovascular**	Arrhythmia, e.g., atrial fibrillation	Hypertension	Palpitations/tachycardia

## Appendix H

**Table A6:** Challenge responses—all elimination diets (strict, moderate, and simple).

Compounds	DBPC		DBPC and Food		Food		All Challenges	
	Patients (n = 3326)	% Positive	Patients (n = 1830)	% Positive	Patients (n = 2429)	% Positive	Patients (n = 4963)	% Positive
Amines	1599	47.22	621	65.86	1197	61.24	3417	55.52
Antioxidant	2120	39.25	70	71.43	373	40.75	2563	40.34
Baker’s yeast	254	35.43					254	35.43
Brewer’s yeast	1368	35.89					1368	35.89
Colors	2741	45.57	82	63.41	411	45.99	3234	46.07
Glutamate	2253	53.93	111	55.86	468	56.84	2832	54.48
Gluten and wheat	755	17.35	1235	58.14	1165	57.42	3155	48.11
Green cordial	133	34.59					133	34.59
Milk and lactose	1160	17.41	1214	56.92	1203	57.36	3577	44.25
Nitrate	2152	42.80	62	61.29	259	36.29	2473	42.58
Preservatives	2892	54.29	172	71.51	318	60.06	3382	55.71
Propionate	1760	41.53	72	59.72	364	40.93	2196	42.03
Salicylate	2195	59.54	657	64.23	1400	65.14	4252	62.11
Soy					337	61.13	337	61.13
Starch	1453	19.20					1453	19.20
Sucrose	1866	20.10					1866	20.10

## Appendix I

**Table A7:** Challenge responses—strict, moderate, or simple elimination diet.

Compounds	Strict Elimination Diet		Moderate Elimination Diet		Simple Elimination Diet		All Elimination Diets	
	Patients (n = 3782)	% Positive	Patients (n = 1066)	% Positive	Patients (n = 115)	% Positive	Patients (n = 4963)	% Positive
Amines	2822	55.42	518	55.98	77	55.84	3417	55.52
Antioxidant	2142	40.94	368	37.23	53	37.74	2563	40.34
Baker’s yeast	120	35.00	132	36.36	2	0.00	254	35.43
Brewer’s yeast	782	36.83	576	34.72	10	30.00	1368	35.89
Colors	2373	47.53	800	41.63	61	47.54	3234	46.07
Glutamate	2364	53.81	405	58.02	63	57.14	2832	54.48
Gluten and wheat	2672	48.80	421	45.61	62	35.48	3155	48.11
Green cordial	128	32.81	5	80.00			133	34.59
Milk and lactose	2691	48.90	825	29.09	61	44.26	3577	44.25
Nitrate	2072	41.51	355	49.58	46	36.96	2473	42.58
Preservatives	2502	55.20	819	57.39	61	54.10	3382	55.71
Propionate	1927	42.81	219	38.36	50	28.00	2196	42.03
Salicylate	3185	61.63	975	63.28	92	66.30	4252	62.11
Soy	280	57.86	46	78.26	11	72.73	337	61.13
Starch	1254	19.94	178	13.48	21	23.81	1453	19.20
Sucrose	1576	20.56	264	18.18	26	11.54	1866	20.10
